# Supplementary material for: Real-time DNA barcoding in a rainforest using nanopore sequencing: opportunities for rapid biodiversity assessments and local capacity building
Source: Gigascience. 2018 Apr 2;7(4):giy033. doi: 10.1093/gigascience/giy033 (PMC5905381; doi:10.1093/gigascience/giy033)
Supplement: GIGA-D-17-00345_Revision_1.pdf [file giy033_giga-d-17-00345_revision_1.pdf]

GigaScience

Real-time DNA barcoding in a rainforest using nanopore sequencing: opportunities for rapid biodiversity assessments and local capacity building

--Manuscript Draft--

**Manuscript Number:**	GIGA-D-17-00345R1
**Full Title:**	Real-time DNA barcoding in a rainforest using nanopore sequencing: opportunities for rapid biodiversity assessments and local capacity building
**Article Type:**	Research
**Funding Information:**	National Geographic Society (W412-15) — Mr Aaron Pomerantz
**Abstract:**	Background<br>Advancements in portable scientific instruments provide promising avenues to expedite field work in order to understand the diverse array of organisms that inhabit our planet. Here, we tested the feasibility for in situ molecular analyses of endemic fauna using a portable laboratory fitting within a single backpack, in one of the world's most imperiled biodiversity hotspots: the Ecuadorian Chocó rainforest. We utilized portable equipment, including the MinION nanopore sequencer (Oxford Nanopore Technologies) and the miniPCR (miniPCR), to perform DNA extraction, PCR amplification and real-time DNA barcoding of reptile specimens in the field.<br><br>Findings<br>We demonstrate that nanopore sequencing can be implemented in a remote tropical forest to quickly and accurately identify species using DNA barcoding, as we generated consensus sequences for species resolution with an accuracy of >99% in less than 24 hours after collecting specimens. The flexibility of our mobile laboratory further allowed us to generate sequence information at Universidad Tecnológica Indoamérica in Quito for rare, endangered, and undescribed species. This includes the recently re-discovered Jambato toad, which was thought to be extinct for 28 years. Sequences generated on the MinION required as little as 30 reads to achieve high accuracy relative to Sanger sequencing and with further multiplexing of samples, nanopore sequencing can become a cost-effective approach for rapid and portable DNA barcoding.<br><br>Conclusions<br>Overall, we establish how mobile laboratories and nanopore sequencing can help to accelerate species identification in remote areas to aid in conservation efforts and be applied to research facilities in developing countries. This opens up possibilities for biodiversity research by promoting local research capacity building, teaching laymen and students about the environment, tackling wildlife crime or by promoting conservation via research focused eco-tourism.
**Corresponding Author:**	Aaron Pomerantz<br>University of California Berkeley<br>UNITED STATES
**Corresponding Author Secondary Information:**	
**Corresponding Author's Institution:**	University of California Berkeley
**Corresponding Author's Secondary Institution:**	
**First Author:**	Aaron Pomerantz
**First Author Secondary Information:**	
**Order of Authors:**	Aaron Pomerantz
	Nicolás Peñafiel
	Alejandro Arteaga

	Lucas Bustamante
	Frank Pichardo
	Luis A Coloma
	César Barrio-Amorós
	David Salazar-Valenzuela
	Stefan Prost
**Order of Authors Secondary Information:**	
**Response to Reviewers:**	Reviewer reports:

Reviewer #1: The authors show us the possibility of real time DNA barcoding by utilizing a few portable laboratory fittings, especially the MinION sequencing platform, which is one of several sequencers that is small enough to be portable for field work. As the authors may aware that several studies have already published in respect to such interests, e. g. Michele et al, Plos One, 2017, on site DNA barcoding by nanopore sequencing.
In addition, the authors may want to conduct some extra analyses and show more details of their results to reach several of their main conclusions. For example:

1. It asked a lot of work to figure out how many samples on earth have been proceeded in current study, which of them have been processed in field? Have all of them conducted PCR aiming at 3 targeted amplicons? All the information can be easily and succinctly demonstrated in one table.

Thank you for the suggestion, we have added a supplementary table that includes all samples from the two runs. This should make it much easier for readers to see what sequencing was performed.

2. By subsampling analysis, the authors tried to say a minimum 30 reads are enough to produce reliable barcode sequences, however, the cytb has got > 30 reads while failed to achieve the final consensus sequence. The other markers can also conduct subsampling of 30 reads at least. In addition, the high throughput sequencing platforms are more sensitive and can generate reads for PCR amplicons albeit no clear band via gel electrophoresis. So, the authors should offer more evidences to say that "none of the reads actually belongs to CytB".

We blasted all reads and added the results to the manuscript. The analysis showed that we only retrieved 0 to ~3 reads for cytB for all samples. We think that the PCR settings, while working well for 16S, did not perform as well for cytB, and have discussed this in the text.

3. The sequencing and Bioinformatics section in supplementary information contains quite a lot valuable information that can help readers better understand current study, the authors may want to move some of the key info to main text. The authors may want to provide details in the form of a table regarding to, for example, how many reads obtained for each marker of each species, the number left after quality filtering, their corresponding length distribution, et al.

We moved the supplementary sections into the main manuscript, and shortened some sections to reduce the length and make it easier to read. We also added % of endogenous DNA barcode reads and % of adapters to the Supplementary table.

4. The authors tried to attribute the higher error rate of ND4 to contamination. Nanopore reads have extremely high error rate, especially for 1D reads, so it won't be a surprise for me if the reads got a top hit to another taxonomic group on NCBI. So, it is better to blast the ND4 reads of another species, e.g. Gecko 2 in your study, to NCBI to see what you can get before reaching the conclusion of contamination. BTW, I suppose you constructed 1D library rather than 2D, please
provide such info in your text.

We blasted ND4 for all species against NCBI, we moved that information from the

Powered by Editorial Manager® and ProduXion Manager® from Aries Systems Corporation

Supplementary to the main manuscript. As can be seen in the new Supplementary table, the Gecko 1 ND4 reads have a much lower % of endogenous ND4 reads when compared to the viper sample (10% compared to 70%).

5. ANGSD -dofasta algorithm would not introduce indel to references, which can be one of the reasons why some of your reference-based consensus sequences contained more errors. The authors may want to compare the references, angsd-based consensus and their corresponding sanger sequences to see whether the discrepancies between each other will engender such bias.

In many cases we found the differences to be insertions of >2 bp compared to the Sanger sequence. In general, mapping approaches show a reference bias, especially for longer indels, but we did not find any of those in our sequenced samples.

6. Fig. 2: the 2 options share the same "read mapping to consensus" that can be merged. Plus, it would be better to provide more details of the denovo method, for example, did you choose the assemblies containing the highest number of reads as candidate consensus? If so, one of the subsampling set 4 (1000 reads, table S3) of gecko 1 got its assembly with only 1 read, what's your criteria for the following selection? And the authors also mentioned that "While the CytB de novo assembly did not succeed (no two reads assembled together)". Did the authors adopt exact same criteria all through?

We changed Supplementary Figure 2. We also added the requested info to the methods.

7. The second sequencing run at UTI generated quite a few adapter dimmers, how about that in the first run? Have you checked that? The information provided in Fig. S1 is quite bewildering, what does the length represent for, 710 bp and 54 bp? Also, what does the red rectangle represent for? Plus, what's the length of adapters, can they be filtered out in the very first step.

We removed Supplementary Figure 1, and added the info to the new Supplementary table. We used filtering using size to establish the % of adapters. So, yes it could be used for filtering, but since the adapter sequences are removed in the processing anyway, it should not matter whether they are removed or not.

8. When the authors talked about cost, not all your amplicons have been successfully sequenced, so $45 per barcode is far from correct estimation. and higher demultiplexing (>300) means less reads generated for each index, which will inevitably lead to higher error rate. The authors may want to tone down this part. In addition, several studies have tried to get individual barcoding at low cost using Miseq and Hiseq platform, you may want to add them in your citation, for example,
Shokralla S, Porter TM, Gibson JF et al. Massively parallel multiplex DNA sequencing for specimen identification using an Illumina MiSeq platform. Sci Rep 2015;5.

Meier R, Wong W, Srivathsan A et al. $1 DNA barcodes for reconstructing complex phenomes and finding rare species in specimen-rich samples. Cladistics 2016;32(1):100-10.

Liu, S., Yang, C., Zhou, C., & Zhou. Filling reference gaps via assembling DNA barcodes using high-throughput sequencing - moving toward barcoding the world. GigaScience, 2017; 1-8.

We have incorporated this additional information to the manuscript. Since we only sequenced a small subset of the total bases of what the MinION can produce there is no issue with low coverage for multiplexing > 300bp. New flow cells reliably produce > 10Gb of output, so comparable output to the MiSeq. We added information on Illumina barcoding to the text.

Lines 195 - 196: it would be better to provide the length of targeted amplions here.
Line 227: What's CS control?
Line 292: Fig. S4 ?
Line 473: please provide ratio at the same time

Powered by Editorial Manager® and ProduXion Manager® from Aries Systems Corporation

	Additional suggestions/questions addressed in text.<br><br>Reviewer #2: Pomerantz et al describe the use of portable nanopore sequencing to assess biodiversity and identify animal species. The paper describes a consequential advance in that portable biodiversity assessment offers a powerful tool for conservation, research and education. I consider the approach of the authors rigourous and the presentation of data and advance made clear.<br><br>My major concern is that the paper spends approximately 250 out of the 600 lines in the main body of the manuscript discussing the broader implications of the work described, rather than the work itself. Evidently, this is an important element of a discussion section, but the narrative developed from ca. line 350 towers over the actual work performed. Many areas of this narrative are speculative: an important role of the discussion is to evaluate the work performed itself, placing it into context, rather than flagging all the possible areas of potential use, or cost implications (which will likely change rapidly) I would strongly recommend that the results and discussion are seperated, and that the discussion is condensed significantly, to strengthen its focus on the results, their limitations and interpretations - before a briefer subsection of the discussion deals with the broader interest areas. This is likely to necessitate a substantial rewrite of the results/discussion section.<br><br>We have moved the results from the Supplementary to the main text, thank you for the suggestion. We have also condensed parts of the text, including in the introduction and discussion.<br><br>Figure 1 is of contextual interest only and is not required to understand the advance presented. It should be removed or merged with Figure 2, which is generally illustrative of the research process.<br><br>Genbank data is listed as pending - thanks for making at available for review.<br><br>Minor comments.<br>L116: Minor point: Not necessarily a standard laptop computer. The specification of laptop according to Oxford Nanopore Technologies is very high, constraining accessiblity.<br>L121: Snowdonia National Park, Wales<br>L123-126: this can be updated to include bacterial ID on the ISS.<br>L278: Should this read Results & Discussion?<br><br>Additional suggestions have been incorporated in the text.
**Additional Information:**	
**Question**	**Response**
Are you submitting this manuscript to a special series or article collection?	No
**Experimental design and statistics**<br><br>Full details of the experimental design and statistical methods used should be given in the Methods section, as detailed in our Minimum Standards Reporting Checklist. Information essential to interpreting the data presented should be made available in the figure legends.<br><br>Have you included all the information requested in your manuscript?	Yes

**Resources**<br><br>A description of all resources used, including antibodies, cell lines, animals and software tools, with enough information to allow them to be uniquely identified, should be included in the Methods section. Authors are strongly encouraged to cite Research Resource Identifiers (RRIDs) for antibodies, model organisms and tools, where possible.<br><br>Have you included the information requested as detailed in our Minimum Standards Reporting Checklist?	Yes
**Availability of data and materials**<br><br>All datasets and code on which the conclusions of the paper rely must be either included in your submission or deposited in publicly available repositories (where available and ethically appropriate), referencing such data using a unique identifier in the references and in the "Availability of Data and Materials" section of your manuscript.<br><br>Have you have met the above requirement as detailed in our Minimum Standards Reporting Checklist?	Yes

Manuscript Click here to download Manuscript Gigascience DNA barcoding rainforest 2_13_17 Final.docx

Real-time DNA barcoding in a rainforest using nanopore sequencing: opportunities for rapid biodiversity assessments and local capacity building

Aaron Pomerantz[1,*], Nicolás Peñafiel[2], Alejandro Arteaga[3–5], Lucas Bustamante[5], Frank Pichardo[5], Luis A. Coloma[6], César L. Barrio-Amorós[7], David Salazar-Valenzuela[2], Stefan Prost [1,8,*]

[1] Department of Integrative Biology, University of California, Berkeley, CA, USA

[2] Centro de Investigación de la Biodiversidad y Cambio Climático (BioCamb) e Ingeniería en Biodiversidad y Recursos Genéticos, Facultad de Ciencias de Medio Ambiente, Universidad Tecnológica Indoamérica, Machala y Sabanilla, Quito, Ecuador

[3] *Richard Gilder Graduate School, American Museum of Natural History, New York, USA*

[4] *Department of Herpetology, American Museum of Natural History, New York, USA*

[5] Tropical Herping, Quito, Ecuador

[6] Centro Jambatu de Investigación y Conservación de Anfibios, Fundación Otonga, Quito, Ecuador

[7] Doc Frog Expeditions, Uvita, Costa Rica

[8] Program for Conservation Genomics, Department of Biology, Stanford University, Stanford, CA, USA

* Corresponding authors: Pomerantz_aaron@berkeley.edu (A. Pomerantz) and stprost@stanford.edu (S. Prost)

Aaron Pomerantz: pomerantz_aaron@berkeley.edu; Nicolás Peñafiel: nicolaspenafielloaiza@gmail.com; Alejandro Arteaga: aarteaga@amnh.org; Lucas Bustamante: lucmat21@gmail.com; Frank Pichardo: frankpichardo@gmail.com; Luis A. Coloma: coloma.l@gmail.com; César L. Barrio-Amorós: cesarlba@yahoo.com; David Salazar-Valenzuela: davidsalazarv@gmail.com; Stefan Prost: stefan.prost@berkeley.edu

Abstract

Background

Advancements in portable scientific instruments provide promising avenues to expedite field work in order to understand the diverse array of organisms that inhabit our planet. Here, we tested the feasibility for *in situ* molecular analyses of endemic fauna using a portable laboratory fitting within a single backpack, in one of the world's most imperiled biodiversity hotspots: the Ecuadorian Chocó rainforest. We utilized portable equipment, including the MinION nanopore sequencer (Oxford Nanopore Technologies) and the miniPCR (miniPCR), to perform DNA extraction, PCR amplification and real-time DNA barcoding of reptile specimens in the field.

Findings

We demonstrate that nanopore sequencing can be implemented in a remote tropical forest to quickly and accurately identify species using DNA barcoding, as we generated consensus sequences for species resolution with an accuracy of >99% in less than 24 hours after collecting specimens. The flexibility of our mobile laboratory further allowed

us to generate sequence information at Universidad Tecnológica Indoamérica in Quito for rare, endangered, and undescribed species. This includes the recently re-discovered Jambato toad, which was thought to be extinct for 28 years. Sequences generated on the MinION required as little as 30 reads to achieve high accuracy relative to Sanger sequencing and with further multiplexing of samples, nanopore sequencing can become a cost-effective approach for rapid and portable DNA barcoding.

Conclusions

Overall, we establish how mobile laboratories and nanopore sequencing can help to accelerate species identification in remote areas to aid in conservation efforts and be applied to research facilities in developing countries. This opens up possibilities for biodiversity research by promoting local research capacity building, teaching laymen and students about the environment, tackling wildlife crime or by promoting conservation via research focused eco-tourism.

Keywords

Nanopore sequencing, portable, DNA barcoding, biodiversity, field-based, real-time

Data Description

Background

Biodiversity is defined as the variety of life found on Earth, including variation in genes, species, and ecosystems. While about 1.9 million species have been described to date,

there are an estimated 5-30 million species in total on the planet, with most of the diversity contained within tropical rainforests [1], [2], [3]. For instance, Ecuador, despite its small size of 283,561 km² (roughly 1.5% of South America), is one of the most biologically diverse countries in the world [4], [5]. Biodiversity is fundamentally important to natural and agro-ecosystems; it provides humans with an array of foods and materials, contributes to medical discoveries, furnishes the economy, and supports ecological services that make life on our planet possible [6]. Today species are going extinct at an accelerated rate because of environmental changes caused by human activities including habitat loss, spread of non-native species, pollution, and climate change [7], [8]. All of these threats have put a serious strain on the diversity of species on Earth.

In the past decade, an ever-growing body of readily accessible knowledge, coupled with new tools in molecular genetics and bioinformatics, have resulted in species being described with greater accuracy, in greater detail, and with additional information to morphological differences. As a result of this increase in quality and content, desirable as it is, the actual process of species description has become slower, while the rate at which species are being lost to extinction has become faster. For many groups of animals, species delimitation can be challenging using solely morphological characteristics [9], [10], and can be improved by incorporating molecular data [11], [12]. This is relevant for the conservation of threatened animals because programs or laws can be implemented more effectively when the existence of a species or population is formally described. DNA barcoding, which is a diagnostic technique that utilizes short conserved DNA sequences, has become a popular tool for a variety of studies including

species identification and molecular phylogenetic inference [13], [14], [15]. Ongoing initiatives, such as 'Barcode of Life' (www.barcodeoflife.org), seek to identify species and create large-scale reference databases via diagnostic DNA sequences using a standardized approach to accelerate taxonomic progress.

While projects utilizing standard molecular markers have grown in popularity in the last decade, a fundamental challenge remains in transporting biological material to a site that can carry out the DNA sequencing. Furthermore, complex and overwhelming regulations can impede biological research in biodiverse countries, and can make it challenging to export material out of the country of origin [16], [17]. Additionally, many research institutions in developing parts of the world do not have access to conventional sequencing technologies within the country, further limiting identification options. This is the case for Ecuador, where most laboratories ship their samples internationally to be sequenced, often creating a delay of weeks to months between tissue collection and the availability of the sequence data. Performing genetic analyses on site or at a nearby facility within the country can help to avoid project delays and decrease the risk of sample quality decline associated with extensive transport. Now it has become possible to take portable lab equipment to remote regions, perform *in situ* experiments, and obtain genetic information relevant for biological studies and conservation policies in real-time.

Portable Sequencing

The MinION (Oxford Nanopore Technologies) is a recently developed nanopore-based DNA sequencing platform. This technology has several advantages over traditional

sequencing technologies, including long-read output, low initial startup costs relative to other commercial sequencers, portability, and rapid real-time analysis (reviewed by [18], [19]). Due to its small size (10 x 3.2 x 2 cm), light weight (90 grams) and ease of power and data transfer (a single USB connection to a standard laptop computer), the MinION has emerged as a valuable tool for portable sequencing projects. This device has been applied in remote sites outside of conventional labs including West Africa to monitor the 2014-2015 Ebola outbreak [20] and Brazil for Zika virus outbreak surveillance [21], [22]. It has also been applied in the Antarctic to sequence microbial communities [23], [24], in Tanzania to sequence frog DNA [25], and in Snowdonia National Park, Wales, for shotgun genomic sequencing of closely-related plant species [26]. The MinION has even been run aboard the International Space Station to evaluate performance off-Earth [27], however, the sequencing runs were performed using DNA libraries pre-prepared in a standard laboratory environment, whereas preparing samples outside of a lab with limited infrastructure presents additional challenges. Indeed, nanopore sequencing appears to hold promise for a variety of molecular experiments in the field.

Scientists have mused over the possibility of a portable method for DNA barcoding for over a decade [28], [15] and in this study our goal was to determine if the steps involved in barcoding, including real-time sequencing with the MinION, could be carried out entirely during a field expedition. We specifically targeted DNA barcodes with existing reference databases because they are the standard approach in molecular biodiversity studies, and allowed us to rapidly produce genetic data for the identification of several animal taxa by multiplexing. Our field site was situated in a remote tropical rainforest and did not offer the commodities of a sophisticated laboratory environment,

including consistent power sources or internet access. We assessed the feasibility for *in situ* genetic sequencing of reptiles and amphibians for rapid species identification, using a portable laboratory fitting within a single backpack, at one of the world's most imperiled biodiversity hotspots, the Ecuadorian Chocó rainforest (Fig. 1). We demonstrate that portable DNA amplicon sequencing with the MinION allows rapid, accurate, and efficient determination at the species level under remote tropical environmental conditions, as well as quick turnaround time for DNA barcodes of undescribed and threatened species at a research facility within the country.

Analyses

Site, sampling, digital photos, tissue collection

We performed all field-based research in the Canandé Reserve (Fig. 1, 0.52993 N, 79.03541 W, 594 m), a 2000 ha. protected area, owned by Jocotoco Foundation (http://www.fjocotoco.org/canandeacute1.html) in Esmeraldas province, northwestern Ecuador. The reserve is located in the Chocó ecoregion and is approximately 6 hours by car, depending on road conditions, from the city of Quito. The majority of organisms sampled in this study were located by space-constrained visual examination of ground-level substrates [1]. The remaining individuals were detected by turning over logs, rocks, and other surface objects. All specimens included in the genetic analyses were morphologically identified based on [2] and [3]. The sample (a tadpole, CJ 7191) of *Atelopus ignescens* was provided by the Museum of Centro Jambatu, Ecuador and was preserved in ethanol 95%. We took vouchers for all samples collected and processed in

the field. These were deposited at the Museo de Zoología of the Universidad Tecnológica Indoamérica (MZUTI 5375 *Bothriechis schlegelii*, MZUTI 5383 *Lepidoblepharis* aff. *grandis.* (Gecko 1), MZUTI 5384 *Lepidoblepharis* aff. *buchwaldi.* (Gecko 2)).

Portable laboratory equipment and set-up

The main items for portable laboratory equipment included the following: two MinION devices, a USB 3.0 cable, three SpotON flow cells (R9.5, Oxford Nanopore Technologies (ONT)), one miniPCR thermocycler (miniPCR), and a benchtop centrifuge (USA Scientific), as well as standard laboratory pipettes and sample racks (Fig. 2, Supplementary Figure 3). The MinKNOW offline software (ONT) required for operation of the MinION was installed and ran on a Windows Vaio Sony laptop with an external SSD drive (VisionTek, 240GB). All heat block and temperature cycling steps were performed using the miniPCR machine, which is a portable thermo-cycler weighing 0.45 kg. The miniPCR was programmed via an application on the laptop and powered by an external battery (PowerAdd). The total amount of equipment could fit in one carry-on backpack; a full list of laboratory hardware is provided as Supplementary Table 1. Reagents for sequencing required frozen transport from the US, which was attained by use of packaging with cold packs in a Styrofoam box and was later transferred to a plastic cool box with further cold packs upon arrival to Quito, Ecuador. MinION flow cells require storage at +2-8°C and were therefore transferred in a food storage container with chilled cold packs. At the field site, reagents and supplies were stored inside a local refrigerator and freezer.

Molecular techniques

Genomic DNA was extracted from fresh blood or tissue samples stored in 95% ethanol using either the DNeasy Blood & Tissue Kit (Qiagen, Hilden, Germany) according to manufacturer's protocol and eluted in 100 µl ddH2O or a modified salt precipitation method based on the Puregene DNA purification kit (Gentra Systems) that involved cellular lysis with SDS and proteinase K, protein precipitation using guanidine isothiocyanate, and DNA precipitation by isopropanol. Tools for manipulating and lysing tissues were sterilized with a flame in between processing samples. We amplified the following mitochondrial DNA fragments: 16S gene using primers 16Sar-L and 16Sbr-H-R from [4], CytB gene using primers L14910 and H16064 developed by [5], and the gene coding for subunit 4 of the NADH dehydrogenase with primers ND4 developed by [6]. All PCR primers contained universal tailed sequences for the Oxford Nanopore Technologies barcoding kit (Supplementary Table 2). We used the ONT PCR Barcoding Kit that allows up to 12 different libraries (barcodes 1-12) to be combined and loaded onto a single flow cell at the same time. PCR reactions contained approximately 1 µl of PCR product, 2.5 µl 10X PCR buffer, 1 µl 25mM $MgCl_2$, 200 µM dNTP mix, 0.2 µM of each primer and 0.25 Platinum Taq DNA Polymerase (Thermo Fisher Scientific) in a 25 µL total volume. All samples for the first PCR run were amplified on the same miniPCR under the following settings: initial denaturation: 94°C for 2 minutes, 35 cycles of denaturation at 94°C for 45 seconds, annealing at 56°C for 60 seconds extension for 72°C for 60 seconds, and a final extension of 72°C for 120 seconds. Then a second round of PCR was carried out, including 2 µl of ONT PCR Barcode, 2 µL of first-round

PCR product, 41 µl H20, and 50 µl PCR reaction mix (0.5 µl Taq DNA polymerase, 1 µL dNTP mix, 2 µL MgCl2, 41 µL H2O). The second round of PCR barcode conditions were modified based on ONT protocol for the Platinum Taq polymerase used in this study as follows: initial denaturation at 95°C for 3 minutes, 15 cycles of denaturation at 95°C for 15 seconds, annealing at 62°C for 15 seconds, extension at 72°C for 60 seconds, and final extension at 72°C for 120 seconds. For verification of samples sequenced in the field, PCR products were subsequently cleaned with Exonuclase I and Alkaline Phosphatase (Illustra ExoProStar by GE Healthcare) at the Universidad Tecnológica Indoamérica (UTI) in Quito and sent to Macrogen Inc (Korea) for Sanger sequencing. All PCR products were sequenced on an ABI3730XL sequencer in both forward and reverse directions with the same primers that were used for amplification. The created sequences were deposited in GenBank (and will be available upon publication). All original Sanger and MinION generated consensus sequences can be found in Additional File 1.

MinION sequencing

DNA library preparation was carried out according to the 1D PCR barcoding amplicons SQK-LSK108 protocol (Oxford Nanopore Technologies). Barcode DNA products were pooled with 5 µl of DNA CS (a positive control provided by ONT) and an end-repair was performed (NEB-Next Ultra II End-prep reaction buffer and enzyme mix, New England Biolabs), then purified using AMPure XP beads. Adapter ligation and tethering was then carried out with 20 µl Adapter Mix (ONT) and 50 µl of NEB Blunt/TA ligation Master Mix (New England Biolabs). The adapter ligated DNA library was then purified with AMPure

beads, followed by the addition of Adapter Bead binding buffer (ONT), and finally eluted in 15 µl of Elution Buffer (ONT). Each R9 flow cell was primed with 1000 µl of a mixture of Fuel Mix (Oxford Nanopore Technologies) and nuclease-free water. Twelve microliters of the amplicon library was diluted in 75 µL of running buffer with 35 µL RBF, 25.5 uL LLB, and 2.5 µL nuclease-free water and then added to the flow cell via the SpotON sample port. The "NC_48Hr_sequencing_FLO-MIN107_SQK-LSK108_plus_Basecaller.py" protocol was initiated using the MinION control software, MinKNOW (offline version provided by ONT).

Bioinformatics

The commands used can be found in the Supplementary Materials and Methods section.

To retrieve the nucleotide sequences from raw signal data generated by the MinKNOW software, we used Albacore 1.2.5 (https://github.com/dvera/albacore) for base calling and de-multiplexing of the ONT barcodes. The FAST5 files were then converted to fastq files using Nanopolish [7]; https://github.com/jts/nanopolish). We then filtered the raw reads for quality (score of >13) and read length (> 200bp) using Nanofilt (https://github.com/wdecoster/nanofilt), and generated consensus sequences using both reference-based mapping and *de novo* assembly. For the reference-based mapping we used BWA 0.7.15 [8]; https://github.com/lh3/bwa/releases) to align the reads to the reference, samtools 1.3 [9] to process the mapping file, and ANGSD [10], to call the consensus sequence. The *de novo* assembly of each amplicon was carried out using Canu [11], https://canu.readthedocs.io), with parameters fitting for our application. Given

that we used short amplicons for the assembly we set the minimum read length to 200bp and the minimum overlap to 50bp. We subsequently extracted the consensus sequences using tgStoreDump. After the consensus calling (for both methods) we mapped the reads back to the consensus sequence (using BWA mem and samtools as described above) and polished the sequencing using Nanopolish [7]. Adapters were removed using Cutadapt [12]. The consensi were then aligned to the Sanger sequences of the same amplicons to investigate the quality of the consensus sequences generated from MinION reads using SeaView [13] and AliView [14]. Sanger sequencing reads were edited and assembled using Geneious R10 software [15] and mapping files inspected by eye using Tablet [16].

We further tested the impact of coverage on the consensus accuracy by randomly subsampling three sets of 30, 100, 300 and 1,000 reads, respectively for the eyelash palm pitviper and gecko 1. Subsampling was performed with famas (https://github.com/andreas-wilm/famas). These sets were assembled *de novo* and processed using the same approach we used for the full data sets (see above).

We then created species alignments for all barcodes (using sequences obtained from Genbank; accession numbers can be found in the phylogenetic tree reconstructions in the Supplementary material). We inferred the best substitution model using jModelTest [17] and reconstructed their phylogenetic trees using the maximum likelihood approach implemented in Mega 5 [18] with 1,000 bootstrap replicates (for bioinformatics workflow see Fig. 3). The output tree files including the Genbank Accession Numbers are provided in the supplementary material.

Results

On July 11, 2017, we arrived at the field site at approximately 1500 hours and collected reptile and amphibian samples from 2000 to 2300 hours. Next, back at the field station, we extracted DNA and performed PCR amplification for 16S, CytB, and ND4 genes. On July 12, the PCR barcodes were pooled, the library was prepared, and then sequencing was initiated at approximately 1600 hours on a flow cell using the offline MinKNOW software, generating 16,663 reads after approximately two hours. The MinKNOW software was then paused in order to assess the reads generated. Within 24 hours of collecting reptiles and amphibians in the Ecuadorian Chocó, we successfully generated consensus sequences for 16S and ND4 genes of an eyelash palm pitviper (*Bothriechis schlegelii*) and 16S for the dwarf gecko (*Lepidoblepharis* sp.; gecko 1). The CytB gene was not successfully sequenced, which was later confirmed at UTI's lab by lack of PCR product on a gel (Supplementary Table 3, Supplementary Figure 4). The field-generated sequence data was analyzed that evening on a laptop using a number of open source and custom-developed bioinformatic workflows (see Materials and Methods). Phylogenetic trees generated using the nanopore sequences and previously generated reference database yielded accurate species identification (Fig. 3 and Fig. 4).

Upon returning to UTI's lab in Quito, we created one additional DNA barcode library with new samples. With our remaining flow cell, we were interested in quickly generating genetic information for (a) additional specimens that were collected during our field expedition (gecko 2), (b) undescribed snake species collected the week before our expedition (Genera: *Dipsas* and *Sibon*), (c) an endangered species that would have

been difficult to export out of the country (Jambato toad), (d) a rare species lacking molecular data (Guayaquil blind snake), and (e) combinations of barcoded samples through multiplexing (for the eyelash palm pitviper and gecko 1).

Initially, this second sequencing run appeared to perform well. However, after using Albacore to demultiplex the reads, we determined the adapter ligation enzyme likely degraded because the output primarily consisted of adapter sequences (Supplementary Figure 1, Supplementary Table 1). Nevertheless, we were able to generate consensus sequences for 16S of the Jambato toad, the two *Dipsas* species, the dwarf gecko, and the Guayaquil blind snake (Fig. 3 and Fig. 4).

The pore count of the flow cells appeared to be unaffected by travel conditions, as indicated by the multiplexer (MUX) scan, an ONT program that performs a quality check by assessing flow cell active pore count. The first run in the field had an initial MUX scan of 478, 357, 177, and 31, for a total of 1,043 active pores and after approximately two hours of sequencing the flow cell generated 16,484 reads. The second flow cell ran at UTI had a MUX scan of 508, 448, 277, and 84, for total of 1,317 active pores and the run produced 21,636 reads within two hours. This is notable since this run was performed 8 days after arriving in Ecuador and the flow cell was stored at suboptimal conditions on site and during travel. The presence or absence of PCR product and size was later determined by gel electrophoresis and quantified by a Quantus Fluorometer (Promega) at UTI. Amplification for 16S and ND4 was successful for all samples, but amplification of CytB was unsuccessful, perhaps due to suboptimal PCR settings, as samples were run concurrently due to the limitation and time-constraint of having only one miniPCR machine available (Supplementary Figure 4).

While the ONT protocol calls for equimolar ratios of pooled PCR product, we did not have an accurate way of quantifying DNA in the field and as such had an overrepresentation of 16S sequences, likely due to PCR bias. On future field expeditions, an inexpensive device such as the bluegel DNA electrophoresis (produced by miniPCR) can be used to assess DNA and PCR products.

Sequencing and Bioinformatics

Eyelash Palm Pitviper (Bothriechis schlegelii)

The eyelash palm pitviper (*B. schlegelii*) is an iconic venomous pitviper species found in mesic forests of Central and northwestern South America [3]. One individual was captured on the evening of the 11th of July 2017 and sequenced on the MinION the following evening. We obtained 3,696 reads for the 16S fragment, 65 reads for CytB, and 94 for ND4. The 16S reads showed an average length of 655bp including the sequencing adapters. The best contig created by Canu was based on 55 reads, to which 3,695 reads mapped for the polishing step. The consensus sequence was 501bp and showed a 100% nucleotide match to the respective Sanger sequence. For this species, we did not find any differences between the *de novo* and the reference-based mapping consensus sequences (generated by mapping against a reference from the same species). The individual clusters with all other *B. schlegelii* and *B. supraciliaris* (considered by some authors to be conspecific with *B. schlegelii*) sequences in the phylogenetic tree (Fig. 4A). While the CytB *de novo* assembly did not succeed (no two reads assembled together), the best supported contig for ND4 (864bp) was based on 50

sequences and achieved an accuracy of 99.4% after polishing (using 95 reads that mapped to the *de novo* consensus).

Dwarf Geckos (Genus: Lepidoblepharis)

Dwarf geckos (genus: *Lepidoblepharis*) are small bodied leaf litter geckos found in Central and South America. Dwarf geckos can be difficult to identify in the field and it is suspected that there are several cryptic species within this genus in Ecuador. We captured two individuals on the evening of the 11th of July 2017, and because the two geckos differed in the shape and size of the dorsal scales (Fig. 3B) and were difficult to confidently identify by morphological characters, we decided to investigate them further with DNA barcoding.

Gecko 1 (*Lepidoblepharis aff. grandis)*

Gecko 1 was included in the first sequencing run in the field. We obtained 4,834 reads for the 16S fragment, 63 reads for CytB, and 76 for ND4. The consensus sequence (522bp) for this individual showed a 100% nucleotide match to the respective Sanger sequence. We then performed reference-based mapping using *L. xanthostigma* (Genbank accession: KP845170) as a reference and the resulting consensus had 99.4% accuracy. We found three insertions compared to the Sanger and the *de novo* consensus sequences (position 302: G and 350-351: AA). Next we attempted assemblies for CytB and ND4. While the assembly for the CytB reads failed, we were able to assemble the ND4 reads. However, the polished consensus sequence showed a relatively high error rate compared to the Sanger sequence (92.1% accuracy). We then

blasted all ND4 reads against NCBI. For ND4 we found 8 sequences to blast to ND4 from squamates, 4 to 16S (3 to a viper and 1 to a gecko), 3 to the positive control, 10 very short hits (negligible hits), and 46 to find no blast hit. Interestingly, while only 8 reads were hits for ND4 from squamates, 72 reads mapped to the consensus of the *de novo* assembly. The higher error rate can thus be explained by the fact that contaminant reads were used to assemble and correct consensus. The *de novo* assembled consensus showed an accuracy of 91.7% compared to 92.1% for the polished sequence.

Gecko 2 (*Lepidoblepharis aff. buchwaldi*)

Gecko 2 was included in the second sequencing run at UTI. We generated 325 reads (for more information see discussion on the possible issue with the adapter ligation enzyme). After filtering for read quality and assembly, we found the best contig to be supported by 30 reads. Out of the 325 barcoded reads, we found 308 to map to the consensus. After running Nanopolish, we found it to match 98.4% to the Sanger sequence. All of the observed differences were indels (mostly 1 bp, but also one 4 bp indel; positions: 15, 23, 217 and 250-253, respectively, Fig. 4B). Positions 15 and 23 show an A in the reference, which is not found in the nanopore consensus (filtered or unfiltered, and polished or not polished). Position 217 is a C in the Sanger reference. None of the consensi for the nanopore data showed the C. This error can potentially be explained as it lies within a 6 bp cytosine homopolymer (see Lu et al., 2016). Interestingly, we saw only a 1bp mismatch instead of the 4bp indel at position 250-253 in the filtered, but not polished nanopore consensus sequence. After polishing all

sequences (filtered or unfiltered) showed the 4bp indel. We next applied reference based mapping (same protocol and reference as for gecko 1). The resulting consensus sequence showed an accuracy of 97.9%. Phylogenetic tree reconstruction shows that gecko 1 and gecko 2 are clearly two distinct species (see Fig. 4B).

Jambato toad (*Atelopus ignescens*)

Laboratory processing and sequencing for *Atelopus ignescens* was carried out in the lab at UTI using a preserved tadpole sample. We obtained 503 reads for this species. The best supported *de novo* assembled contig was based on 56 reads. We then mapped the reads back to this contig for the polishing step, which resulted in 491 mapped reads. However, while the total coverage was 434x for the segment, the average coverage was only 212x. The discrepancy can be explained by a high percentage of reads that exclusively consisted of adapter sequences (probably caused by inefficient adapter ligation; see Discussion section; Supplementary Figure 1). The resulting sequence fits 100% to the respective Sanger sequence (Fig. 4C). We next used the reference-based approach to construct a consensus sequence, using *Atelopus hoogmoedi* (Genbank accession: EU672974) as a reference and the consensus achieved an accuracy of 100% after polishing. The phylogenetic tree reconstruction clusters our sequence with samples described as *A.* sp. *aff. ignescens*.

Guayaquil blind snake (Trilepida guayaquilensis)

The Guayaquil blind snake (*Trilepida guayaquilensis*) belongs to the family of Slender blind snakes (Leptotyphlopidae). This family is found in North and South America, Asia,

and Africa. They are fossorial snakes adapted to life underground. The Guayaquil blind snake was only known from one individual described in 1970 and is endemic to Ecuador [19]. For a second specimen collected by Jose Vieira on March 03, 2016 at Pacoche, province of Manabi, Ecuador (S1.0677 W80.88169 323m), we obtained 756 sequences. However, many of those reads were adapter sequences. The Canu *de novo* assembled sequence was generated from 16 reads. We then mapped 740 reads back to this consensus. After polishing the consensus sequence matched 100% of the Sanger generated sequence (Fig. 5A; 516bp consensus length). We further investigated the accuracy of reference based mapping for this species. We used *Trilepida macrolepis* (Genbank accession: GQ469225) as a reference, which is suspected to be a close relative of *T. guayaquilensis.* However, the resulting consensus sequence had a lower accuracy (97.7%) compared to the *de novo* assembled consensus (100%). Our sequence is sister to the clade comprising *Trilepida macrolepis* and all *Rena* species in the phylogenetic tree.

Dipsas snakes (Genus: Dipsas)

Dipsas are non-venomous New World colubrid snakes that are found in Central and South America (Cadle 2005). Here we included two specimens collected one week prior to our expedition.

Dipsas oreas (MZUTI 5418)

We generated 779 reads for *Dipsas oreas* (MZUTI5418). The best supported contig of the Canu *de novo* assembly (498bp consensus length) was based on 59 reads and

matched the corresponding Sanger sequence to 99% after polishing (Fig. 5B). Three out of 5 mismatches were indels in poly-A stretches (position: 185, 287, 411). The remaining two mistmachtes are a C to G at position 469 and a T to A at position 489 for the nanopore compared to the Sanger sequence. Interestingly, the reference-based consensus sequence (using *Dipsas* sp., GenBank accession: KX283341 as a reference) matched the Sanger sequence to 99.4% after polishing. We generated 816 reads for the CytB barcode. However, *de novo* assembly was not successful as only three reads blasted to CytB. However, the lengths of the hits were insignificant. Two sequences blasted to 16S, one blasted to a Dipsadine snake and one to *Atelopus*. One read belonged to the positive control and 53 showed insignificantly short hits.

Dipsas oreas (MZUTI 5415)

We generated 487 reads for *Dipsas* (MZUTI 5415). Sequences with a quality score of >13 were retained resulting in 193 sequences. The best supported contig of the Canu *de novo* assembly was based on 59 reads (498bp consensus length). After polishing the consensus sequence matched the corresponding Sanger sequence to 98.9% (Fig. 5B). The first two mismatches are typical nanopore errors, namely indels in poly-A stretches (positions: 287, 411). The nanopore sequence shows an insertion of a single G compared to the Sanger sequence as position 431. The last mismatch is a three base pair deletion compared to the Sanger sequence (positions: 451-453). The reference-based consensus (using *Dipsas* sp., GenBank accession: KX283341 as a reference) achieved a 98.4% match after polishing. We generated 1,077 reads for the CytB barcode. Again, *de novo* assembly was not successful as only four reads actually

belonged to CytB. Four sequences belonged to the positive control, seven to 16S (four blasted to Colubridae, and three to squamates), one to a Viperidae microsatellite, and 51 gave insignificantly short hits. The two *Dipsas* specimens clustered together in the phylogeny. They are sister to the clade comprising *D. neivai* and *D. variegata*. However, this part of the phylogeny shows low support (bootstraps < 50).

Sibon sp. (Genus: Sibon)

Sibon snakes are found in northern South America, Central America and Mexico [20]. We generated 339 reads for the 16S barcode of this species. However, we were not able to create a consensus sequence for this barcode, as almost all the reads were adapter sequences (all but 11 reads). Furthermore, we generated 1,425 reads for the CytB barcode but were not able to create a consensus sequence.

Subsampling

We further investigated the read depth needed to call accurate consensus sequences using our approach. We used the eyelash palm pitviper and gecko 1 to test subsampling schemes, since we obtained thousands of reads for these samples. We randomly subsampled to 30, 100, 300 and 1,000 reads (in three replicates; see Supplementary Table 4). For the eyelash palm pitviper we achieved accuracies ranging from 99.4% to 99.8% using only 30 reads, 99.6% to 100% using 100 reads, 99.8% for 300 reads and 99.8% to 100% for 1,000 reads. For gecko 1 we achieved even better accuracy overall, with 30 reads ranging from 99.4% to 99.8%, 100 reads from 99.8% to

100%, all 300 reads sets achieved an accuracy of 100% and for 1,000 reads all but one set (99.8%) achieved 100% accuracy.

Multiplexing

We further sequenced multiplexed barcodes (16S and ND4) for the eyelash palm pitviper and gecko 1. However, we did not obtain reads for this sample from sequencing run 2, most likely due to the adapter ligation issues. We thus generated artificial multiplexes for the eyelash palm pitviper pooling random sets of 1,000 16S reads with all 96 ND4 reads to investigate the performance of the *de novo* assembly using multiplexed samples. We assembled the reads *de novo* and processed them using the same approach as discussed above. In all three cases, we found the first two contigs of the canu run to be 16S and ND4 contigs. After polishing the 16S consensus sequences achieved a 99.8% accuracy (all three assemblies showed a deletion in a stretch of four T's compared to the Sanger sequence) and the ND4 sequences a 99.4% accuracy. All errors, but one (which shows a T compared to the C in the Sanger sequence), in ND4 are deletions in homopolymer stretches.

Discussion

Performance in the field

Our objective was to employ a portable laboratory in a rainforest to quickly identify endemic species with DNA barcoding (Fig. 2). Our protocols resulted in successful DNA extraction, PCR amplification, nanopore sequencing, and barcode assembly, with a

turnaround time of less than 24 hours. We observed that the MinION sequencing platform performed well in the field after extended travel, indicating the potential for nanopore-based sequencing on future field expeditions. Although we demonstrate that the successful molecular identification of organisms in a remote tropical environment is possible, challenges with molecular work in the field remain. Our field site was provided with inconsistent electrical power, but still allowed us to use a conventional small centrifuge for several steps of DNA extraction and to power a refrigerator for storage of flow cells and some of the reagents, although temperatures were likely suboptimal. Lack of electrical supply can impede adequate storage of temperature-sensitive reagents for extended periods of time. Our experiments were performed during a relatively short field trial, with 10 days being the longest time period that reagents were kept at inconsistent freezing temperatures. It is uncertain how well nanopore kit reagents or flow cell integrity would endure over longer periods without consistent cooling temperatures, and we suspect the adapter ligation enzyme was compromised during our second nanopore run, as demultiplexing led to a majority of barcode adapters in each folder (Supplementary Table 3). While the MinION sequencer fits in the palm of a hand and needs only a USB outlet to function, bioinformatic analyses can be hampered under remote field conditions, because internet access, large amounts of data storage, and long periods of time are often required for such analytical tasks. In our study, utilizing short DNA fragments with a relatively small number of samples for barcoding allowed us to perform all bioinformatic analyses in the field, but larger data outputs may require additional storage and more computational resources.

Implications for conservation and biodiversity assessments

Tropical rainforests, such as the Ecuadorian Chocó, are often rich in biodiversity, as well as species of conservation concern. The Chocó biogeographical region is one of the world's 25 biodiversity hotspots [29] and several studies have identified the Chocó region of western Colombia and Ecuador as a global conservation priority [29], [30], [31]. We therefore chose this region for proof of principle *in situ* molecular work to highlight the importance of expediting fieldwork in order to produce genetic information of endemic fauna. Our rapidly obtained DNA barcodes allowed us to accurately identify organisms while in the field. When samples are not required to be exported out of the country to carry out molecular experiments, real-time sequencing information can contribute to more efficient production of biodiversity reports that advise conservation policy, especially in areas of high conservation risk.

Of particular note in this study was the critically endangered harlequin Jambato toad, *Atelopus ignescens*. Although not a denizen of the Chocó rainforests, this Andean toad is a good example to demonstrate how nanopore sequencing can aid in the conservation of critically endangered species. *Atelopus ignescens* was previously presumed extinct (it is currently still listed as "extinct" on IUCN; [32]) and was only recently rediscovered [33]. The last confirmed record of *Atelopus ignescens* dates back to 1988, and this species was presumed to be extinct before one population was rediscovered in 2016, 28 years later. *Atelopus* is a species-rich genus of neotropical toads containing 96 species, most of which are possibly extinct or endangered. In Ecuador there are 11 species of *Atelopus* that are Critically Endangered (tagged as Possibly extinct; [34]). Extinctions of *Atelopus* (and other anurans) are beyond control

and are increasingly exacerbated by a combination of factors including habitat loss, climate change and pathogens [35], [36], [37]. For the many endangered species that are protected by international laws and treaties, sample transport requires permits that can often be difficult to obtain, even when research is expressly aimed at conservation, resulting in project delays that can further compromise sample quality. By working within the country, under permits issued by Ministerio del Ambiente de Ecuador to local institutions, we were able to generate sequence data for the endangered harlequin Jambato toad *Atelopus ignescens* within 24 hours of receiving the tissue, whereas obtaining permits to ship samples internationally in the same time frame would have not been possible. Rapidly identifying the phylogenetic affinity of populations of *Atelopus* toads could speed up conservation efforts for these animals. Namely, a better understanding of the systematics of the group facilitated by real-time sequencing could help establish species limits, geographic distributions, in-situ conservation actions and ex-situ breeding programs.

Species identifications

It is important to note that we do not intend for rapidly-obtained portable sequence information to substitute for standard species description processes. Instead, we aim to demonstrate that obtaining real-time genetic information can have beneficial applications for biologists in the field, such as raising the interesting possibility of promptly identifying new candidate species, information which can be used to adjust fieldwork strategies or sampling efforts. As we have shown, the latter could be especially important with organisms and habitats facing pressing threat. Rapidly

obtaining genetic sequence information in the field can also be useful for a range of other applications, including identifying cryptic species, hybrid zones, immature stages, and species-complexes.

Furthermore, we acknowledge that in most cases multiple loci are needed to reliably infer species position in a phylogenetic tree. DNA barcoding has been shown to hold promise for identification purposes in taxonomically well-sampled clades, but may have limitations or pitfalls in delineating closely related species or in taxonomically understudied groups [38], [39]. However, our aim in this study was to demonstrate that portable sequencing can be used in the field and that the final sequences have an accuracy needed to achieve reliable identification of a specimen. While a recent study has demonstrated a field-based shotgun genome approach with the MinION to identify closely related plant species [26], DNA barcoding already offers a robust reference database for many taxa thanks in part to global barcoding initiatives (the current Barcode of Life Data System contains 4,013,927 specimens and 398,087 Barcode Index Numbers http://ibol.org/resources/barcode-library/ as of September 2017).

Finally, while highlighting the value of real-time portable DNA barcoding in this study, we do not wish to downplay the significance of taxonomic experts, who have invaluable specialist knowledge about specific groups of organisms. Even with the advent of molecular diagnostic techniques to describe and discover species, placing organisms within a phylogenetic context based on a solid taxonomic foundation is necessary. An integrative approach utilizing molecular data and morphological taxonomy can lead to greater insight of biological and ecological questions [40]. As noted by Bik, 2017, *“There is much to gain and little to lose by deeply integrating*

morphological taxonomy with high-throughput sequencing and computational workflows."

Bioinformatic challenges

While we were able to show that nanopore sequencing results in high quality DNA barcode sequences, some challenges during the read processing remain. To our knowledge, no software solution specifically designed to assemble DNA barcodes from long read technologies is available. Here, we created our own pipeline (Supplementary Figure 2). This required changing the settings for Canu [41], a whole genome *de novo* assembler (see Materials and Methods in the Supplementary Information; and discussion below). However, software geared towards the specifics of assembling DNA barcodes from long read data would be beneficial to make the bioinformatics analysis easier and more widely applicable.

We were also interested in investigating the minimum coverage needed to create reliable consensus sequences. Therefore, we used different subsampling schemes. Overall, a coverage of 30 reads achieved an accuracy of 99.4 - 99.8%. With 100x read coverage almost all assemblies were 100% accurate, indicating that an excessive number of reads is not needed to produce high quality consensus sequences. Furthermore, we applied Nanopolish to all consensus sequences. This tool has been shown to be very effective at correcting typical nanopore errors, such as homopolymer errors [42], [43]. As can be seen in section "Post-Nanopolish assembly identity" in [43], accuracy of the resulting consensus increases significantly after polishing. While, we did not measure the improvement in accuracy in our study, we did notice a high accuracy

after polishing. However, as can be seen in Fig. 4B, nanopolish is not always able to accurately correct homopolymer stretches.

We further tested reference-based mapping versus *de novo* assembly, because a reference-based mapping approach may introduce bias, making it possible to miss indels. Overall, we see that consensus sequences generated using reference-based mapping have slightly lower accuracy. However, in two cases (the eyelash palm pitviper and the Jambato toad) an accuracy of 100% was achieved with reference-based mapping. Interestingly, in the case of *Dipsas* sp. (MZUTI 5418), reference-based mapping resulted in a slightly better accuracy than the *de novo* approach (99.4% compared to 99%). However, in general, we recommend the use of a *de novo* assembly approach as this method can be applied even if no reference sequence is available and generally produced more accurate sequences. An alternative approach would be to generate consensus sequences by aligning the individual reads for each barcode to one another, which would not be affected by a reference bias. This method is implemented in the freely available software tool Allele Wrangler (https://github.com/transplantation-immunology/allele-wrangler/). However, at the time of submission this tool picks the first read as the pseudo reference, which can lead to errors in the consensus if this read is of particularly low quality or an incorrect (contaminant) sequence. Future developments might establish this method as an alternative to *de novo* assembly algorithms, which are typically written for larger genomes (e.g. the minimum genome size in Canu is 1000bp) and can have issues with assemblies where the consensus sequence is roughly the size of the input reads (*personal communications* Adam Phillippy).

Each of our two runs showed a very high number of reads not assigned to any barcode sequence after de-multiplexing with Albacore 1.2.5 (7,780 and 14,272 for the first and second sequencing run, respectively). In order to investigate whether these reads belong to the target DNA barcodes but did not get assigned to sequencing barcodes, or if they constitute other sequences, we generated two references (one for each sequencing run) comprising all consensi found within each individual sequencing run. We then mapped all reads not assigned to barcodes back to the reference. We were able to map 2,874 and 4,997 reads to the reference for the first and the second sequencing run, respectively, which shows that a high number of reads might be usable if more efficient de-multiplexing algorithms become available. Here we used Albacore 1.2.5, an ONT software tool, to de-multiplex the sequencing barcodes. This tool is under constant development and thus might offer more efficient de-multiplexing in later versions. Alternatively, 3rd party software tools like npBarcode [44] or Porechop (https://github.com/rrwick/Porechop) can be used.

Cost-effectiveness and local resource development

Next-generation sequencing technologies are constantly evolving, along with their associated costs. Most major next-generation sequencing platforms require considerable initial investment in the sequencers themselves, costing hundreds of thousands of dollars, which is why they are often consolidated to sequencing centers at the institutional level [45]. In this study, we used the ONT starter pack, which currently costs $1000, and includes two flow cells and a library preparation kit (6 library preparations), as well as the ONT 12 barcoding kit which is currently $250 for 6 library

preparations (for a full list of equipment and additional reagents see Supplementary Table 1). Using this setup, each barcode amplicon sequence generated costs approximately $45 (this includes cost for the starter pack, etc; a detailed cost account can be found in the Supplementary material). At this cost, further multiplexing of samples on each flow cell is necessary to achieve a cost-effectiveness for DNA sequencing relative to other commercial options. However, it will likely not be long until much higher multiplexing (>500 samples) becomes achievable on the MinION platform, which would pave the way for MinION-based DNA barcode costs to be reduced to less than $1, similar to advancements achieved in Illumina and PacBio-based pipelines (see [46], [47], [48]). On the contrary, Sanger sequencing from UTI in Ecuador shipped internationally for processing costs approximately $10 per sample, independent of the through-put. Thus, the Oxford Nanopore MinION has the potential to be a cost-effective sequencing option for resource-limited labs, especially in developing countries without access to standard sequencing devices.

The small size and low power requirements of the MinION will likely continue to enable its evolution as a field-deployable DNA sequencing device, opening up new avenues for biological research in areas where the typical laboratory infrastructure for genetic sequencing is unavailable. With some training, in the field molecular analyses could also potentially be performed by students (see [49]) or assistants, providing an opportunity for local teaching and research capacity building, and community involvement via research focused ecotourism or citizen-science projects.

Future outlook

Technological developments in lab equipment and reagent chemistry are increasingly enabling the incorporation of genetic analyses into field projects. Several portable technologies have been used to perform molecular experiments in the field, particularly for disease diagnostics [50], [51]. Advances in lyophilized and room-temperature reagents are also promising for field applications, such as EZ PCR Master Mix [52], and loop-mediated isothermal amplification [53], [54]. A hand-powered centrifuge [55] could also act as substitute for a standard benchtop centrifuge during DNA extraction steps. Automatic devices, such as VolTRAX (a compact microfluidic device designed to automate nanopore library preparation, ONT) and improved library construction methods may offer faster and high-throughput methods for preparing nanopore libraries in the future. As the ONT MinION evolves, it could greatly advance field researchers' capacity to obtain genetic data from wild organisms while in the field. These technologies currently depend on reagents that require freezing, but can be used at field sites with solar or portable freezer options. Faster and more automated sample processing, as well as cost reductions, are needed for adoption in low-income settings.

Beyond short PCR-based amplicons aimed at species identification, other exciting potential applications of nanopore sequencing in the field include sequencing of entire mitochondria from gDNA samples [56] or via long-range PCR, shotgun genome sequencing [26], analysis of environmental DNA [57], [24], sequencing of direct RNA [58], [59] or cDNA to rapidly profile transcriptomes ([60], and pathogen diagnostics and monitoring (such as chytrid fungus; [61]). Rapid portable sequencing can also be applied to wildlife crime to perform species identification of animals affected by illegal

trafficking, as well as serve to aid in early detection of invasive species threatening local biodiversity and agriculture, and emerging infectious diseases.

Potential implications

While we live in a period of amazing technological change, biodiversity and ecosystem health are decreasing worldwide. Portable sequencing will not be a silver bullet for conservation biology, but it can be a powerful tool to more efficiently obtain information about the diversity of life on our planet. This is particularly important for many biodiversity hotpots, such as tropical rainforests like the Ecuadorian Chocó, which are often under high risk of habitat loss. Here we show that portable DNA barcoding with the MinION sequencer allows rapid, accurate, and efficient determination at the species level under remote and tropical environmental conditions. We also demonstrate that portable sequencing can allow nimble use of rapidly generating data for endangered, rare, and undescribed species at nearby facilities within the country. As portable technologies develop further, this method has the potential to broaden the utility of biological field analyses including real-time species identification, cryptic species discovery, biodiversity conservation reports, pathogen detection, and environmental studies.

Competing interests

The authors report no competing interests.

Author contributions

AP and SP designed the project. AP, NP, AA, LB, FP, CB, DSV and SP carried out specimen collection; AP and NP laboratory work; AA, LB, FP, LC, CB and DSV morphological species identification and SP computational analyses. AP, NP, AA, LB, FP, LC, CB, DSV and SP wrote the paper.

Acknowledgements

Fieldwork for this project was made possible with the support of Tropical Herping and Universidad Tecnológica Indoamérica. For granting access to the Canandé reserve, we are grateful to Martin Schaefer of Fundación Jocotoco. Funding for equipment and reagents was supported by the National Geographic Society / Waitt grant (W412-15). Travel funding for Stefan Prost was provided by the Program of Conservation Genomics at Stanford University. Laboratory work was carried out at Universidad Tecnológica Indoamérica in Quito. Research and collection permits were issued by the Ministerio del Ambiente de Ecuador (MAE-DNB-CM-2015-0017). The Jambato toad tissue was provided by the Museum of Centro Jambatu under the Ministerio del Ambiente de Ecuador project “Conservation of Ecuadorian amphibian diversity and sustainable use of its genetic resources.” We thank Oxford Nanopore Technologies for providing technical support, making the offline MinKNOW software available, and for providing two free flow cells. We also thank Hitomi Asahara and the UC Berkeley DNA Sequencing Facility for lending the benchtop centrifuge used in this study; Jared Simpson, Sergey Koren and Adam Phillippy for very helpful advice and discussion on the bioinformatic pipeline, and Ellie E. Armstrong for valuable input on the manuscript.

Figures

Figure 1. Site where field-based nanopore research was conducted within the Chocó biogeographical region in Ecuador, which is one of the world's 25 biodiversity hotspots. This area has experienced one of the highest rates of deforestation in the country and is considered a global conservation priority.

Figure 2. Process of nanopore sequencing in the Ecuadorian Chocó rainforest. A) Sampling endemic fauna; eyelash pitviper next to MinION. B) Extraction of blood or tissue samples. C) DNA extraction using the DNeasy kit and benchtop centrifuge, and PCR amplification with the MiniPCR. D) Oxford nanopore library preparation of DNA barcodes. E) Bioinformatic processing of nanopore data in the field. F) Primary equipment used in portable sequencing, left to right: MiniPCR sitting atop Poweradd external battery, MinION plugged into a Windows laptop displaying Geneious Pro software of raw nanopore data.

Figure 3. Species investigated, nucleotide alignments of nanopore and Sanger sequences comparing consensus accuracy, and Maximum Likelihood trees of 16S sequences for: A) Eyelash pitviper, *Bothriechis schlegelii*, B) two species of dwarf gecko, *Lepidoblepharis* sp, and C) the Jambato toad, *Atelopus ignescens.* Red labels in the phylogenetic trees indicate the sequences generated by the MinION.

Figure 4. Species investigated, nucleotide alignments of nanopore and Sanger sequences comparing consensus accuracy, and Maximum Likelihood trees of 16S sequences for: A) Guayaquil blind snake, *Trilepida guayaquilensis* and B) two species *Dipsas* snakes. Red labels in the phylogenetic trees indicate the sequences generated by the MinION.

References

1. Erwin TL. Tropical Forests Their Richness in Coleoptera and Other Arthropod Species. The Coleop Bull. 1982;36:74-75.
2. Stork NE. How many species are there? Biodiv Conserv. 1993;2:215-232.
3. Scheffers BR, Joppa LN, Pimm SL, Laurance WF. What we know and don't know about Earth's missing biodiversity. Trends Ecol Evol. 2012;27:501-510.
4. Sierra R, Campos F, Chamberlin J. Assessing biodiversity conservation priorities: ecosystem risk and representativeness in continental Ecuador. Landscape and Urban Planning. 2002;59:95-110.
5. Cuesta F, Peralvo M, Merino-Viteri A, Bustamante M, Baquero F, et al. Priority areas for biodiversity conservation in mainland Ecuador. Neotrop Biodiv. 2017;3:93-106.
6. Gascon C, Brooks TM, Contreras-MacBeath T, Heard N, Konstant W, et al. The importance and benefits of species. Curr Biol. 2015;25:R431-438.

7. Pimm SL, Jenkins CN, Abell R, Brooks TM, Gittleman JL, et al. The biodiversity of species and their rates of extinction, distribution, and protection. Science. 2014;344:1246752.

8. Ceballos G, Ehrlich PR, Dirzo R. Biological annihilation via the ongoing sixth mass extinction signaled by vertebrate population losses and declines. Proc Natl Acad Sci USA. 2017;114:E6089-E6096.

9. Colonma LA, Duellman WE, Almendariz A, Ron, SR. Five new (extinct?) species of Atelopus (Anura: Bufonidae) from Andean Colom-bia, Ecuador, and Peru. Zootaxa. 2010;2574:1-54.

10. Loetters S, Van der Meijden A, Coloma LA, Boistel R, Cloetens P, et al. Assessing the molecular phylogeny of a near extinct group of vertebrates: the Neotropical harlequin frogs (Bufonidae; Atelopus). System Biodiv. 2011;9:45-57.

11. Barley AJ, White J, Diesmos AC, Brown RM. The challenge of species delimitation at the extremes: diversification without morphological change in philippine sun skinks. Evolution. 2013;67:3556-3572.

12. Carstens BC, Pelletier TA, Reid NM, Satler JD. How to fail at species delimitation. Mol Ecol. 2013;22:4369-4383.

13. Hebert PD, Cywinska A, Ball SL, deWaard JR. Biological identifications through DNA barcodes. Proc Biol Sci. 2003;270:313-321.

14. Hebert PD, Gregory TR. The promise of DNA barcoding for taxonomy. Syst Biol. 2005;54:852-859.

15. Savolainen V, Cowan RS, Vogler AP, Roderick GK, Lane R. Towards writing the encyclopedia of life: an introduction to DNA barcoding. Philos Trans R Soc Lond B Biol Sci. 2005;360:1805-1811.

16. Gilbert N. Biodiversity law could stymie research. Nature Publishing Group. 2010; http://www.nature.com/news/2010/100203/full/463598a.html

17. Fernández F. The greatest impediment to the study of biodiversity in Colombia. Caldasia. 2011;33:3-5.

18. Laver T, Harrison J, O'Neill PA, Moore K, Farbos A, et al. Assessing the performance of the Oxford Nanopore Technologies MinION. Biomol Detect Quantif. 2015;3:1-8.

19. Mikheyev AS, Tin MM. A first look at the Oxford Nanopore MinION sequencer. Mol Ecol Resour. 2014;14:1097-1102.

20. Quick J, Loman NJ, Duraffour S, Simpson JT, Severi E, et al. Real-time, portable genome sequencing for Ebola surveillance. Nature. 2016;530:228-232.

21. Faria NR, Sabino EC, Nunes MR, Alcantara LC, Loman NJ, et al. Mobile real-time surveillance of Zika virus in Brazil. Genome Med. 2016;8:97.

22. Faria NR, Quick J, Claro IM, Theze J, de Jesus JG, et al. Establishment and cryptic transmission of Zika virus in Brazil and the Americas. Nature. 2017;546:406-410.

23. Edwards A, Debbonaire AR, Sattler B, Mur LAJ, Hodson AJ. Extreme metagenomics using nanopore DNA sequencing: a field report from Svalbard, 78 °N. bioRxiv. 2016; https://doi.org/10.1101/073965

24. Johnson SS, Zaikova E, Goerlitz DS, Bai Y, Tighe SW. Real-Time DNA Sequencing in the Antarctic Dry Valleys Using the Oxford Nanopore Sequencer. J Biomol Tech. 2017;28:2-7.

25. Menegon M, Cantaloni C, Rodriguez-Prieto A, Centomo C, Abdelfattah A, Rossato M, Bernardi M, Xumerle L, Loader S, Delledonne M. On site DNA barcoding by nanopore sequencing. PLoS One. 2017;https://doi.org/10.1371/journal.pone.0184741

26. Parker J, Helmstetter AJ, Devey D, Wilkinson T, Papadopulos AST. Field-based species identification of closely-related plants using real-time nanopore sequencing. Sci Rep. 2017;7:8345.

27. Castro-Wallace SL, Chiu CY, John KK, Stahl SE, Rubins KH, et al. Nanopore DNA Sequencing and Genome Assembly on the International Space Station uploaded to BioRxiv. 2016; https://doi.org/10.1101/077651

28. Janzen DH, Hajibabaei M, Burns JM, Hallwachs W, Remigio E, et al. Wedding biodiversity inventory of a large and complex Lepidoptera fauna with DNA barcoding. Philos Trans R Soc Lond B Biol Sci. 2005;360:1835-1845.

29. Myers N, Mittermeier RA, Mittermeier CG, da Fonseca GA, Kent J. Biodiversity hotspots for conservation priorities. Nature. 2000;403:853-858.

30. Dinerstein E. A Conservation assessment of the terrestrial ecoregions of Latin America and the Caribbean. Washington, D.C.: World Bank. 1995;xvii, 129 p. p.

31. Olson DM, Dinerstein E. The Global 200: a representation approach to conserving the Earth’s most biologically valuable ecoregions. Conservation Biology. 1998;12:502-515.

32. Santiago Ron LAC, Stefan Lötters, William Duellman, Martín R. Bustamante, Wilmar Bolívar, Enrique La Marca. 2004 IUCN Red List Atelopus ignescens

33. Coloma L. El Jambato negro del páramo, Atelopus ignescens, resucitó. 2016 IMCiencia.

34. Tapia EE, Coloma LA, Pazmiño-Otamendi G, Peñafiel N. Rediscovery of the nearly extinct longnose harlequin frog Atelopus longirostris (Bufonidae) in Junín, Imbabura, Ecuador. Neotropical Biodiversity. 2017;3:157-167.

35. Kiesecker JM. Global stressors and the global decline of amphibians: tipping the stress immunocompetency axis. Ecological research. 2011;26:897-908.

36. Pounds JA, Coloma LA. Beware the lone killer. Nature Reports Climate Change. 2008;57-59.

37. Lips KR, Diffendorfer J, Mendelson III JR, Sears MW. Riding the wave: reconciling the roles of disease and climate change in amphibian declines. PLoS biology. 2008;6: e72.

38. Elias M, Hill RI, Willmott KR, Dasmahapatra KK, Brower AV, et al. Limited performance of DNA barcoding in a diverse community of tropical butterflies. Proc Biol Sci. 2007;274:2881-2889.

39. Meyer CP, Paulay G. DNA barcoding: error rates based on comprehensive sampling. PLoS Biol. 2005;3: e422.

40. Bik HM. Let’s rise up to unite taxonomy and technology. PLoS Biology. 2017;15: e2002231.

41. Koren S, Walenz BP, Berlin K, Miller JR, Bergman NH, et al. Canu: scalable and accurate long-read assembly via adaptive k-mer weighting and repeat separation. Genome Res. 2017;27:722-736.

42. Loman NJ, Quick J, Simpson JT. A complete bacterial genome assembled de novo using only nanopore sequencing data. Nat Methods. 2015;12:733-735.

43. Wick RR, Judd LM, Gorrie CL, Holt KE (2017) Completing bacterial genome assemblies with multiplex MinION sequencing. bioRxiv. 2017;160614.

44. Nguyen SH, Duarte TDS, Coin LJ, Cao MD. Real-Time Demultiplexing Nanopore Barcoded Sequencing Data With npBarcode. bioRxiv. 2017;134155.

45. Glenn TC. Field guide to next-generation DNA sequencers. Molecular ecology resources. 2011;11: 759-769.

46. Meier R, Wong W, Srivathsan A, Foo M. $1 DNA barcodes for reconstructing complex phenomes and finding rare species in specimen-rich samples. Cladistics 2016;32(1):100-10.

47. Liu S, Yang C, Zhou C, Zhou X. Filling reference gaps via assembling DNA barcodes using high-throughput sequencing - moving toward barcoding the world. GigaScience, 2017; 1-8.

48. Hebert PDN, Brakmann TWA, Prosser SWJ, Ratnasingham S, deWaard JR, Ivanova NV, Janzen DH, Hallwachs W, Naik S, Sones JE, Zakharov EV. A Sequel to Sanger: Amplicon Sequencing That Scales. bioRxiv, doi: https://doi.org/10.1101/191619

49. Zeng, Y., & Martin, CH. Oxford Nanopore sequencing in a research-based undergraduate course. bioRxiv *2017;*227439.

50. Canier L, Khim N, Kim S, Sluydts V, Heng S, et al. An innovative tool for moving malaria PCR detection of parasite reservoir into the field. Malar J. 2013;12:405.

51. Marx V. PCR heads into the field. Nature methods. 2015;12:393-397.

52. Guevara EE, Frankel DC, Ranaivonasy J, Richard AF, Ratsirarson J, et al. A simple, economical protocol for DNA extraction and amplification where there is no lab. Conservation Genetics Resources. 2017;1-7.

53. Centeno-Cuadros A, Abbasi I, Nathan R. Sex determination in the wild: a field application of loop-mediated isothermal amplification successfully determines sex across three raptor species. Mol Ecol Resour. 2017;17:153-160.

54. Howson ELA, Armson B, Madi M, Kasanga CJ, Kandusi S, et al. Evaluation of Two Lyophilized Molecular Assays to Rapidly Detect Foot-and-Mouth Disease Virus Directly from Clinical Samples in Field Settings. Transbound Emerg Dis. 2017;64:861-871.

55. Bhamla MS, Benson B, Chai C, Katsikis G, Johri A, et al. Hand-powered ultralow-cost paper centrifuge. Nature Biomedical Engineering. 2017;1: 0009.

56. Chandler J, Camberis M, Bouchery T, Blaxter M, Le Gros G, et al. Annotated mitochondrial genome with Nanopore R9 signal for Nippostrongylus brasiliensis. F1000Res. 2017;6: 56.

57. Rees HC, Maddison BC, Middleditch DJ, Patmore JR, Gough KC. The detection of aquatic animal species using environmental DNA–a review of eDNA as a survey tool in ecology. Journal of Applied Ecology. 2014;51:1450-1459.

58. Ayub M, Hardwick SW, Luisi BF, Bayley H. Nanopore-based identification of individual nucleotides for direct RNA sequencing. Nano Lett. 2014;13: 6144-6150.

59. Garalde DR, Snell EA, Jachimowicz D, Heron AJ, Bruce M, et al. Highly parallel direct RNA sequenc ing on an array of nanopores bioRxiv. 2016;https://doi.org/10.1101/068809

60. Hargreaves AD, Mulley JF. Assessing the utility of the Oxford Nanopore MinION for snake venom gland cDNA sequencing. PeerJ. 2015;3:e1441.

61. Weldon C, du Preez LH, Hyatt AD, Muller R, Spears R. Origin of the amphibian chytrid fungus. Emerg Infect Dis. 2004;10:2100-2105.

Figure 1 Click here to download Figure Fig1.tif

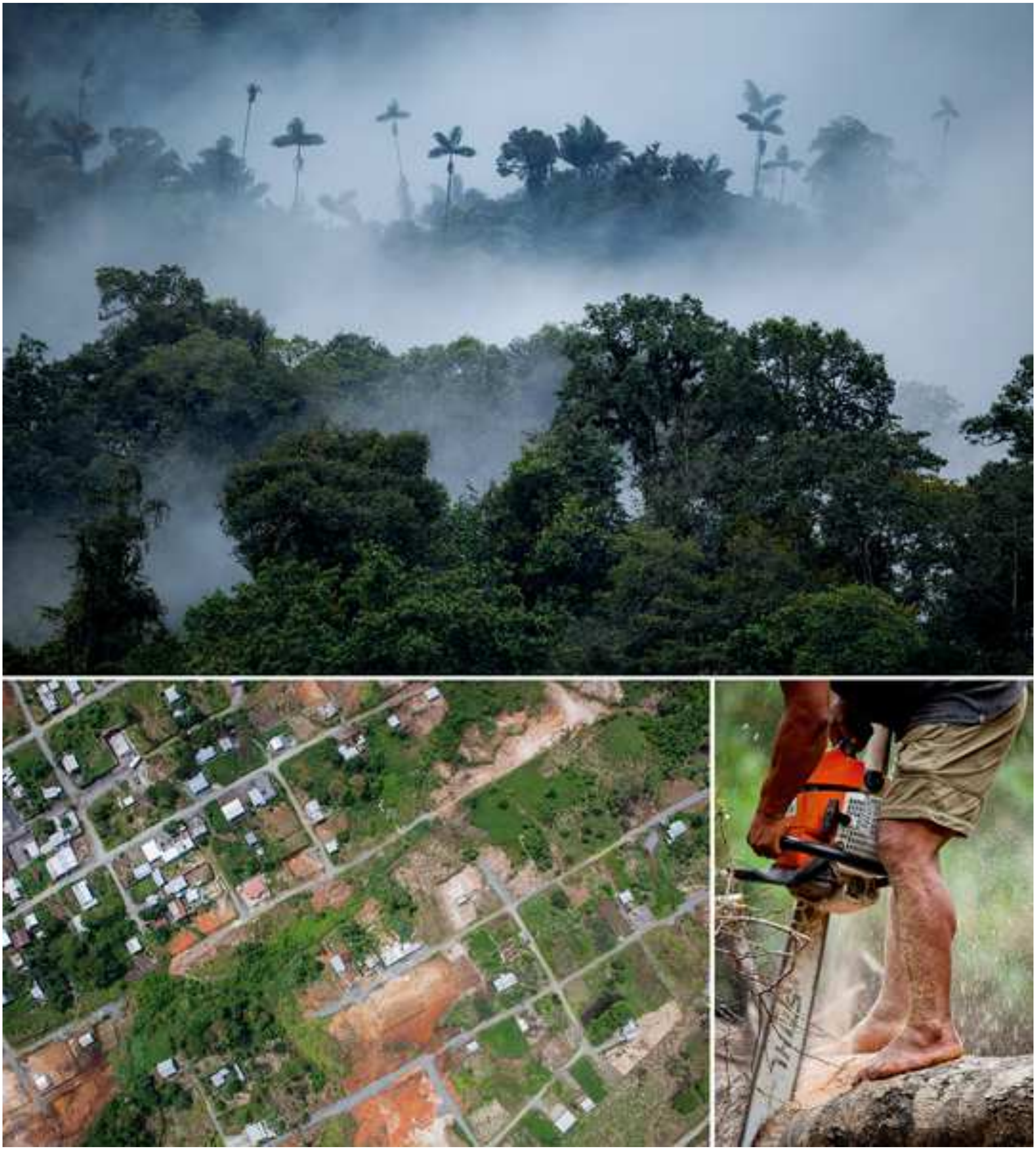

Figure 2 Click here to download Figure Fig2.tif

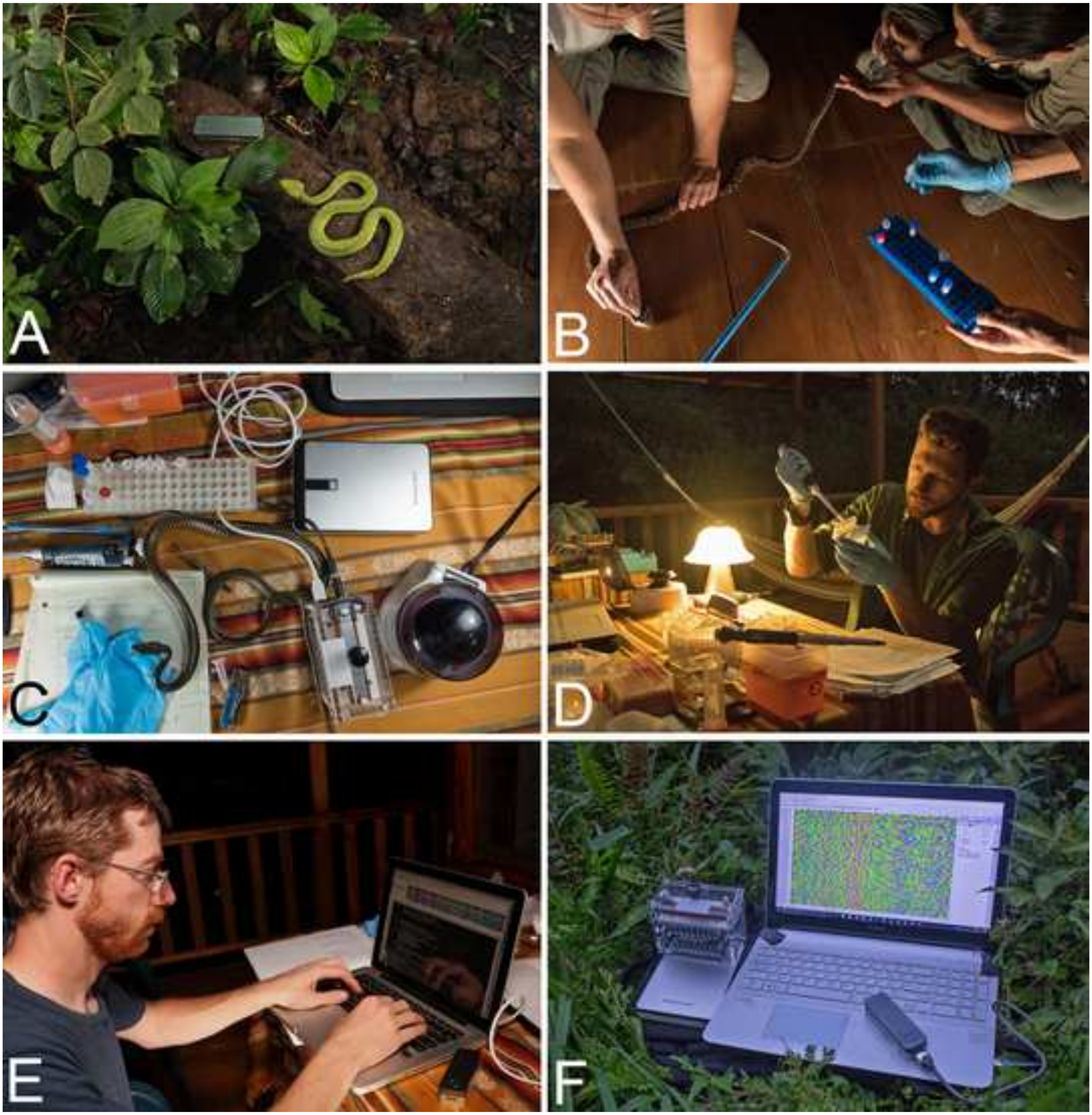

Figure 3

Click here to download Figure Fig3.pdf

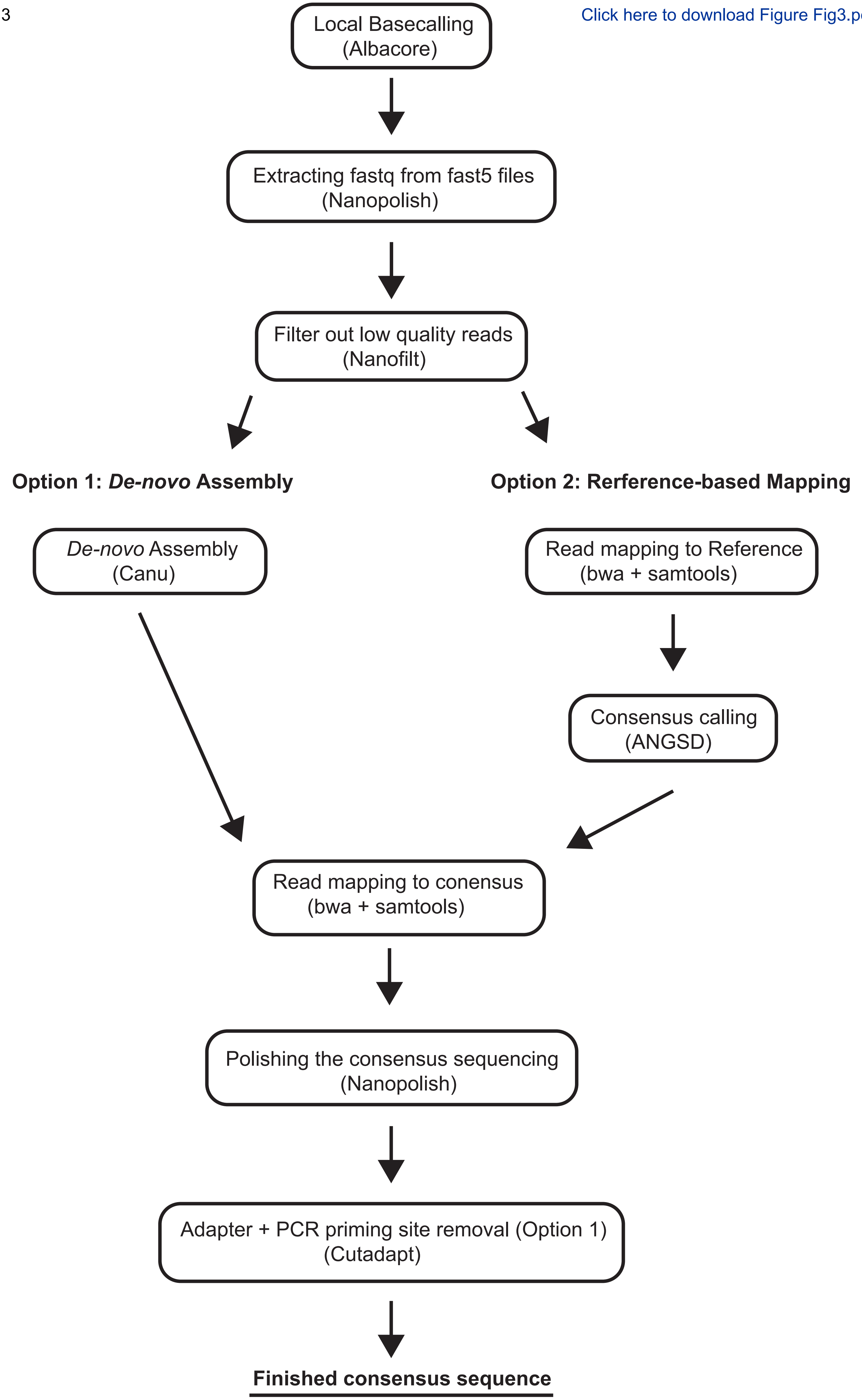

Figure 4

Click here to download Figure Fig4.tif

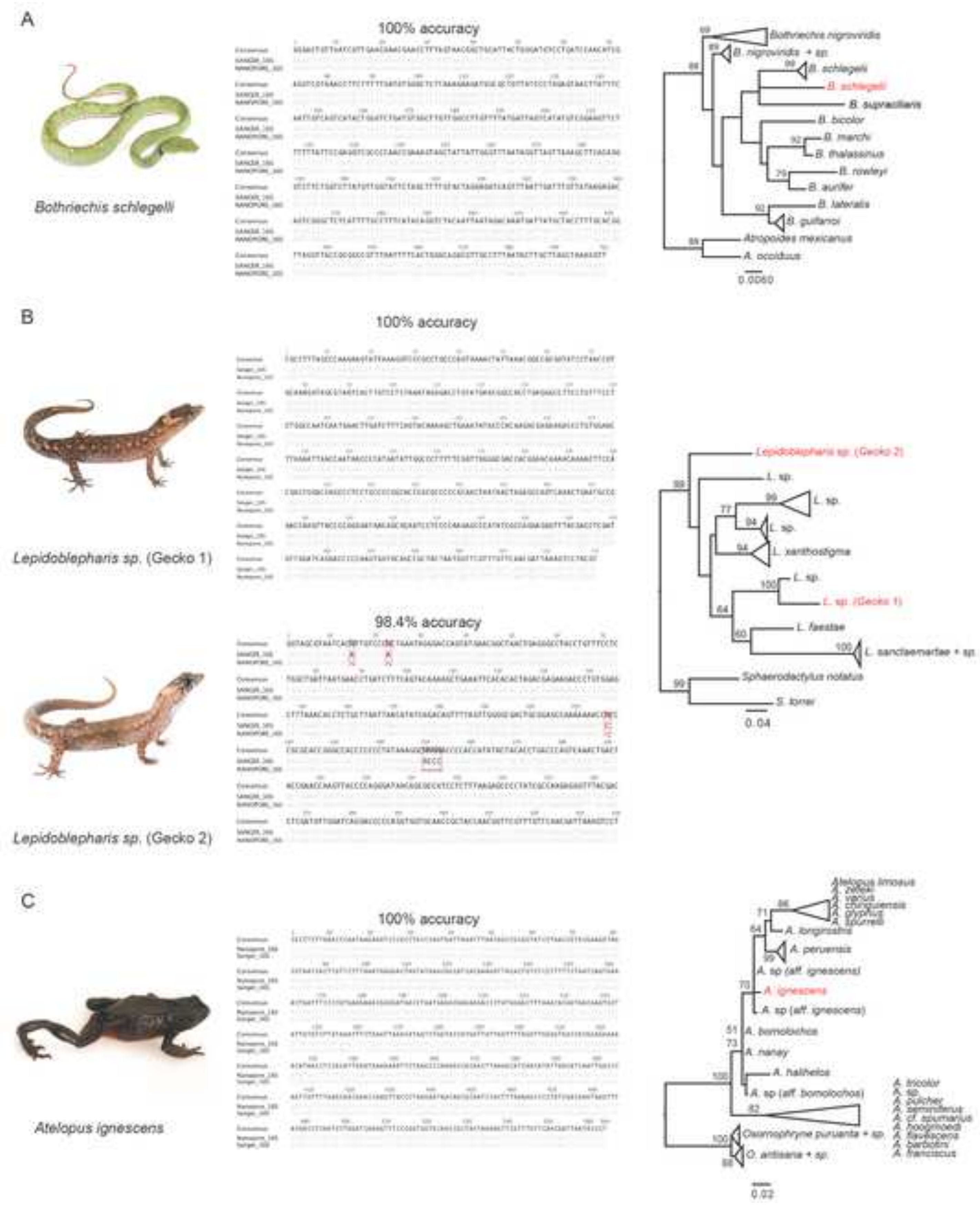

Figure 5

Click here to download Figure Fig5.pdf

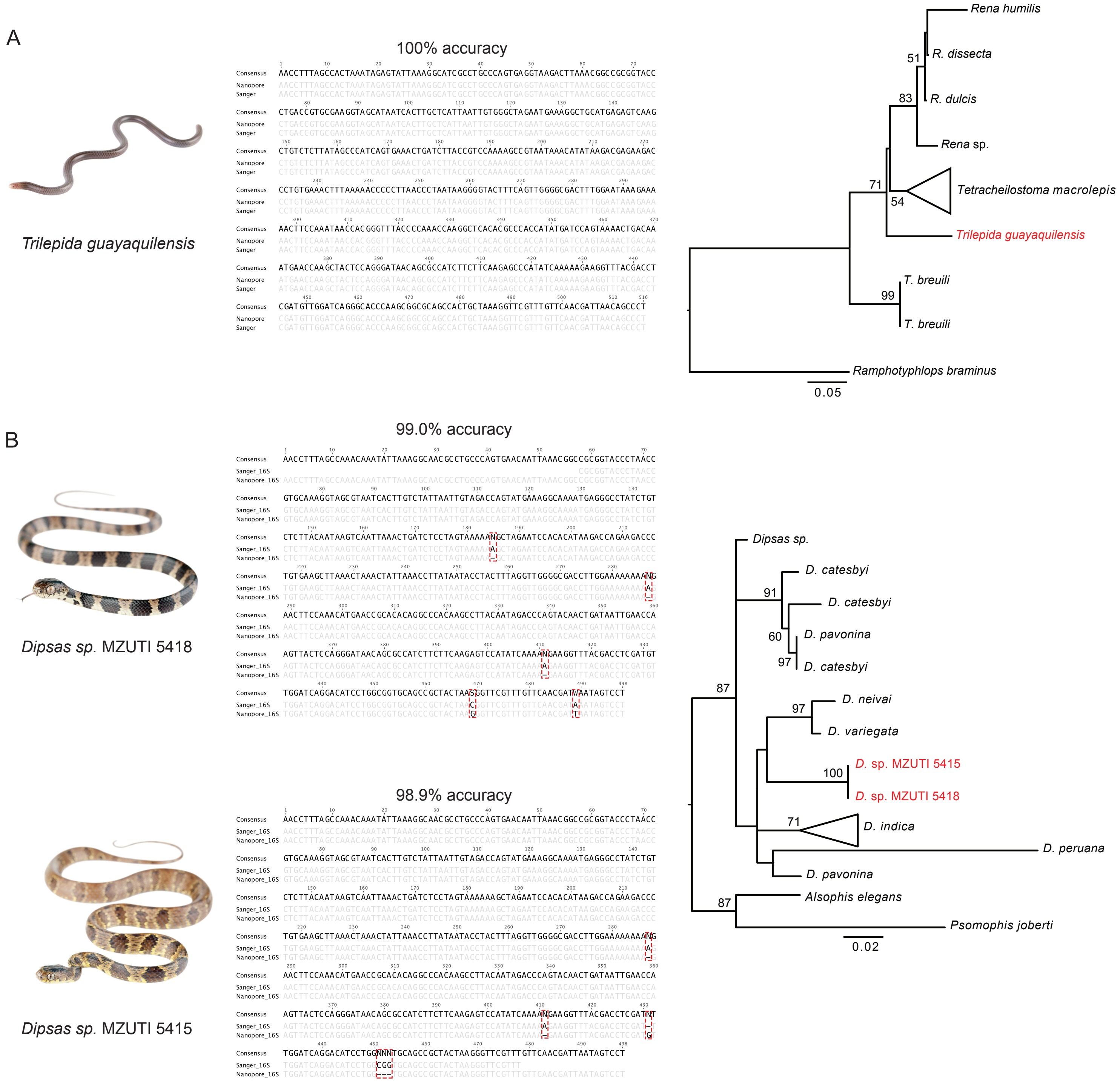

Supplementary Table 1

Click here to access/download
Supplementary Material
Supplementary Table 1.pdf

Supplementary Table 2

Click here to access/download
Supplementary Material
Supplementary Table 2.pdf

Supplementary Figure 1

Click here to access/download
Supplementary Material
Supplementary_Figure_1.jpg

Supplementary Figure 2

Click here to access/download
Supplementary Material
Supplementary_Figure_2.tif

Supplementary Figure 3

Click here to access/download
Supplementary Material
Supplementary_Figure_3.jpg

Supplementary Figure 4

Click here to access/download
Supplementary Material
Supplementary_Figure_4.jpg

Supplementary Table 3

Click here to access/download
Supplementary Material
Supplemental Table 3.pdf

Supplementary Table 4

Click here to access/download
Supplementary Material
Supplementary Table 4.pdf

Click here to access/download
Supplementary Material
SUPPLEMENTARY INFORMATION GigaScience.docx
