# Supplementary material for: Real-time DNA barcoding in a rainforest using nanopore sequencing: opportunities for rapid biodiversity assessments and local capacity building
Source: Gigascience. 2018 Apr 2;7(4):giy033. doi: 10.1093/gigascience/giy033 (PMC5905381; doi:10.1093/gigascience/giy033)
Supplement: Supplemental material [file giy033_supp.zip › Supplemental Table 3.pdf]

Supplemental Table 3: Summary of de-multiplexed reads.

First flow cell run:

Sample name	Number of raw reads	Percent of adapter sequences	Percent of endogenous barcode reads	Average read length
Viper 16S	3696	0.05%	90.1%	654.9 ± 90.0
Viper cytb	65	6.15%	0%	530.3 ± 497
Viper ND4	96	3.12%	70.8%	796 ± 294.8
Dwarf 158 16S	4834	0.01%	84.1%	658.8 ± 65.3
Dwarf 158 cytb	63	9.52%	0%	509.6 ± 247.1
Dwarf 158 ND4	76	6.68%	10.5%	639.7 ± 620.0
Unclassified	7780	5.05%		NA

Second flow cell run:

Sample name	Number raw reads	Percent of adapter sequences	Percent of endogenous barcode reads	Average read length
Dipsas 396 16S	487	30.18%	15.6%	270.1 ± 168.0
Dipsas 396 cytb	1077	34.91%	0%	216.6 ± 183.2
Dipsas 378 16S	779	36.84%	21.6%	294.9 ± 200.3
Dipsas 378 cytb	816	52.94%	0%	192.3 ± 65.3
Dwarf 213 16S	325	48.62%	8%	231.0 ± 129.5
Sibon 16S	339	47.20%	0%	196.2 ± 61.0
Sibon cytb	1425	37.19%	0%	205.6 ± 119.5
Blind snake 16S	756	26.72%	35.8%	386.4 ± 259.7
Atelopus 16S	503	195 (38.77%)	39.6%	378.2 ± 246.7
Unclassified	23049	7380 (33.97%)		NA
