# Supplementary material for: Real-time DNA barcoding in a rainforest using nanopore sequencing: opportunities for rapid biodiversity assessments and local capacity building
Source: Gigascience. 2018 Apr 2;7(4):giy033. doi: 10.1093/gigascience/giy033 (PMC5905381; doi:10.1093/gigascience/giy033)
Supplement: Supplemental material [file giy033_supp.zip › SUPPLEMENTARY INFORMATION GigaScience.docx]

SUPPLEMENTARY INFORMATION

**Supplementary Figures**

**Supplementary Figure 1.** Large portion of adapter sequences contained in demultiplexed barcodes, indicating possible adapter ligation degradation for the second nanopore run at UTI.

**Supplementary Figure 2.** Bioinformatics workflow summarizing the steps performed during nanopore sequencing analysis with either a *de novo* approach (left) or reference-based mapping approach (right), in order to generate a consensus sequences

**Supplementary Figure 3.** Additional images highlighting the portable lab equipment and setup used for nanopore sequencing in Ecuador. A) The handheld MinION DNA sequencer (Oxford Nanopore Technologies). B) miniPCR Thermocycler (miniPCR). C) Mobile setup for DNA extraction and PCR amplification. D) Loading the ONT flow cell in the field. E) Running the MinION using offline MinKNOW software. F) Local collaborator loading the MinION at a nearby research facility, highlighting the opportunity for capacity building and community involvement.

**Supplementary Figure 4.** Gel of PCR product that was produced in the field using the miniPCR and imaged at UTI in Quito. Note that 16S and ND4 from samples amplified but CytB did not.

**Bioinformatics Commands**

***Sequencing and base calling***

The library was sequenced on the MinION platform using an offline MinKnow version without local base calling. At the time our team departed for the field only a Windows MinKnow offline version was available, thus we carried out sequencing and base calling on a Windows laptop. The analysis was subsequently performed using a Linux Ubuntu system. The sequencing was carried out using a MinION R9.5 flow cell. We then used Albacore 1.2.5 for local base calling and demultiplexing.

-i E:\2017_Ecudador_Barcode_7_12_17 -t 2 -s E:\2017_Ecudador_Barcode_7_12_17 -f FLO-MIN107 --kit SQK-LSK108 --recursive --barcoding --output_format fast5

All the following steps were performed on a UNIX based platform (and should work on both MacOSX and Linux). In order to be able to perform sequence polishing we outputted fast5 in Albacore. The fast5 files for each individual barcode were subsequently converted to fastq files using Nanopolish.

nanopolish extract -b albacore --fastq barcode01/ > barcode01_eyelash_palm-pitviper_16S.fastq

Nanopolish also adds a directory path to the fastq header in order to use the fast5 information later in the polishing.

Next, we employed different consensus sequence generation methods, namely reference-based mapping, reference-based mapping using an ONT read as reference and de novo assembly of the individual amplicon sequences.

***Read Filtering***

cat barcode01_eyelash_palm-pitviper_16S.fastq | NanoFilt -q 15 -l 500 > barcode01_eyelash_palm-pitviper_16S_filtered.fastq

***De-novo assembly of the Amplicons (Preferred Option)***

We created the consensus sequence *de novo*, so without the use of a reference sequence. Here we used the genome assembler Canu with adapted parameters for shorter reads. *De-novo* assembly of the amplicons is preferred over reference-based mapping (see below), since it does not introduce any biases by mapping it to a reference, which might have indels compared to the reads.

To perform a *de novo* assembly for each amplicon using canu we run:

canu –p barcode01_eyelash_palm-pitviper_16S_canu –d barcode01_eyelash_palm-pitviper_16S_canu genomeSize=1000 minReadLength=100 minOverlapLength=50 -nanopore-raw barcode01_eyelash_palm-pitviper_16S.fastq

To output the consensus, we ran:

tgStoreDump -T unitigging/barcode01_eyelash_palm-pitviper_16S_canu.ctgStore 2 -G unitigging/ barcode01_eyelash_palm-pitviper_16S_canu.gkpStore -consensus -fasta -tig 1 > barcode01_eyelash_palm-pitviper_16S_canu.bestcontig.fasta

Alternatively, we saved all contigs to a file using:

tgStoreDump -T unitigging/barcode01_eyelash_palm-pitviper_16S_canu.ctgStore 2 -G unitigging/ barcode01_eyelash_palm-pitviper_16S_canu.gkpStore -consensus -fasta > barcode01_eyelash_palm-pitviper_16S_canu.contigs.fasta

We then created consensus sequences the same commands as described below (bwa index, bwa mem, samtools view, samtools sort, samtools index and nanopolish variants). We used the first contig from canu as the reference for the mapping. We further performed mapping against a reference that contained all canu generated contigs to check how many reads map to the different contigs. The first contig always showed the highest number of reads.

In the last step, we removed adapters and the priming sites using cutadapt.

cutadapt -g CGCCTGTTTAYCAAAAACAT...ACGTGATCTGAGTTCAGACCGG -o pitviper_filtered_canu.best.contig_cut.fasta pitviper_filtered_canu.best.contig.fasta

***Standard Reference-based Mapping***

For Option 2 we mapped the reads to a reference sequence downloaded from NCBI (here GenBank Accession KC847257). In the first step, we indexed the reference.

           bwa index KC847257_bothriechis_schlegelii.fasta

We then mapped the reads onto the indexed reference using BWA mem. The mem algorithm was specifically designed for mapping reads to a divergent reference. Here we used bwa 0.7.12, which provides an option –d ont2d, which employs the following filters -k14 -W20 -r10 -A1 -B1 -O1 -E1 -L0 in order to optimize the mapping for Oxford Nanopore read data.

bwa mem -x ont2d KC847257_bothriechis_schlegelii.fasta barcode01_eyelash_palm-pitviper_16S.fastq > barcode01_eyelash_palm-pitviper_16S.sam

Here KC847257_bothriechis_schlegelii.fasta was the reference that we used in the mapping and barcode01_eyelash_palm-pitviper_16S.fastq the read data. In the next step, we used samtools 1.3.1 to convert the mapping in sam format to a bam format, which performs block compression to reduce the file size. Mapping files in bam format are standardly used in downstream analysis.

samtools view –bS barcode01_eyelash_palm-pitviper_16S.sam > barcode01_eyelash_palm-pitviper_16S.bam

We then sorted and indexed the bam file using samtools to allow rapid random access for indexing queries.

samtools sort barcode01_eyelash_palm-pitviper_16S.bam –o barcode01_eyelash_palm-pitviper_16S.srt.bam

samtools index barcode01_eyelash_palm-pitviper_16S.srt.bam

In the next step, we called the consensus sequence using ANGSD v. 0.911-51-g57d0264

angsd –doFasta 3 –doCounts 1 barcode01_eyelash_palm-pitviper_16S.srt.bam –out barcode01_eyelash_palm-pitviper_16S_angsd

The –doFasta 3 option performs consensus calling using highest effective depth (EBD; [21]). The fasta file can be unzipped afterwards using “gzip –d file”.

In the next steps, we mapped all the read data back onto the created consensus sequence to enable consensus polishing using nanopolish (same commands as before: bwa index, bwa mem, samtools view, samtools sort and samtools index). Finally, we created a polished consensus using nanopolish.

nanopolish variants --consensus=barcode01_eyelash_palm-pitviper_16S _cons.fasta --bam barcode01_eyelash_palm-pitviper_16S.srt.bam --genome barcode01_eyelash_palm-pitviper_16S_angsd.fasta --reads barcode01_eyelash_palm-pitviper_16S.fastq

**Calculations for cost in this study, based on expenses as of July, 2017:**

- ONT starter kit: $1000
- ONT 12 barcode kit: $250 for 6 library constructions, therefore each library per run = $41.7
- 2 barcode libraries generated in this study = (41.7 x 2) + $1000 (ONT starter pack) = $1083.4
- Number of barcodes generated by 2 rounds of sequencing: 12 x 2 = 24 barcodes
- $1083.4 / 24 barcodes = $45.1 per barcode

**References**

1. Campbell HW, Christman SP (1982) Field techniques for herpetofaunal community analysis. Herpetological communities: 193-200.

2. Calderon-Espinosa ML, Medina-Rangel GF (2016) A new Lepidoblepharis lizard (Squamata: Sphaerodactylidae) from the Colombian Guyana shield. Zootaxa 4067: 215-232.

3. Campbell JA, Lamar WW, Brodie ED (2004) The venomous reptiles of the Western Hemisphere. Ithaca N.Y.: Comstock Pub. Associates.

4. Palumbi S (1991) Simple fool's guide to PCR.

5. Burbrink FT, Lawson R, Slowinski JB (2000) Mitochondrial DNA phylogeography of the polytypic North American rat snake (Elaphe obsoleta): a critique of the subspecies concept. Evolution 54: 2107-2118.

6. Arèvalo E, Davis SK, Sites Jr JW (1994) Mitochondrial DNA sequence divergence and phylogenetic relationships among eight chromosome races of the Sceloporus grammicus complex (Phrynosomatidae) in central Mexico. Systematic Biology 43: 387-418.

7. Loman NJ, Quick J, Simpson JT (2015) A complete bacterial genome assembled de novo using only nanopore sequencing data. Nat Methods 12: 733-735.

8. Li H, Durbin R (2009) Fast and accurate short read alignment with Burrows–Wheeler transform. Bioinformatics 25: 1754-1760.

9. Li H, Handsaker B, Wysoker A, Fennell T, Ruan J, et al. (2009) The sequence alignment/map format and SAMtools. Bioinformatics 25: 2078-2079.

10. Korneliussen TS, Albrechtsen A, Nielsen R (2014) ANGSD: analysis of next generation sequencing data. BMC bioinformatics 15: 356.

11. Koren S, Walenz BP, Berlin K, Miller JR, Bergman NH, et al. (2017) Canu: scalable and accurate long-read assembly via adaptive k-mer weighting and repeat separation. Genome Res 27: 722-736.

12. Martin M (2011) Cutadapt removes adapter sequences from high-throughput sequencing reads. EMBnet journal 17: pp. 10-12.

13. Gouy M, Guindon S, Gascuel O (2010) SeaView version 4: A multiplatform graphical user interface for sequence alignment and phylogenetic tree building. Mol Biol Evol 27: 221-224.

14. Larsson A (2014) AliView: a fast and lightweight alignment viewer and editor for large datasets. Bioinformatics 30: 3276-3278.

15. Kearse M, Moir R, Wilson A, Stones-Havas S, Cheung M, et al. (2012) Geneious Basic: an integrated and extendable desktop software platform for the organization and analysis of sequence data. Bioinformatics 28: 1647-1649.

16. Milne I, Bayer M, Cardle L, Shaw P, Stephen G, et al. (2009) Tablet—next generation sequence assembly visualization. Bioinformatics 26: 401-402.

17. Posada D (2008) jModelTest: phylogenetic model averaging. Molecular biology and evolution 25: 1253-1256.

18. Tamura K, Peterson D, Peterson N, Stecher G, Nei M, et al. (2011) MEGA5: molecular evolutionary genetics analysis using maximum likelihood, evolutionary distance, and maximum parsimony methods. Mol Biol Evol 28: 2731-2739.

19. Cisneros-Heredia DF (2017) IUCN Red List Trilepida guayaquilensis

20. Fitzinger LJ (1826) Neue classification der Reptilien nach ihren naturlichen Verwandtschaften: Heubner.

21. Wang Y, Lu J, Yu J, Gibbs RA, Yu F (2013) An integrative variant analysis pipeline for accurate genotype/haplotype inference in population NGS data. Genome Research 23: 833-842.
