# Supplementary material for: Real-time DNA barcoding in a rainforest using nanopore sequencing: opportunities for rapid biodiversity assessments and local capacity building
Source: Gigascience. 2018 Apr 2;7(4):giy033. doi: 10.1093/gigascience/giy033 (PMC5905381; doi:10.1093/gigascience/giy033)
Supplement: Supplemental material [file giy033_supp.zip › Supplementary Table 1.pdf]

Supplementary Table 1. List of equipment, consumables, and reagents used for portable nanopore sequencing in Ecuador.

**Equipment**	**Supplier**
Laptop	Windows, MacBook Pro
MinION	Oxford Nanopore Technologies
Flow cell, R9.5	Oxford Nanopore Technologies
miniPCR thermal cycler	miniPCR
External SSD Flash Drive	VisionTek
Benchtop centrifuge	USA Scientific
Pipettes, Gilson (P20, P200, P1000)	ThermoFischer
Magnetic rack	Promega
External battery source	PowerAdd
Gel electrophoresis chamber (MSMINI)	Biocomdirect
300V electrophoresis power source	VWR International
Tube racks for 1.5 mL tubes	-
**Consumables**	
Gloves	-
Pipette tips (P20, P200, P1000)	ThermoFischer
1.5 mL Eppendorf tubes	ThermoFischer
0.2 mL PCR tubes	ThermoFischer

Plastic pestles	-
Polystyrene thermal control box	-
Scissors, tweezers	-
Frozen ice packs	-

**Reagents**	**Experiment**
Extraction buffer	DNA extraction
SDS	
proteinase K	
RnaseA	
isopropanol	
NaCl	
EtOH	
10x PCR buffer	PCR
MgCl2	
dNTP	
Primers	
Taq polymerase	
Nuclease free water	
Illustra Exostar (PCR cleanup)	

Agarose	Gel electrophoresis
TBE	
Ladder	
SYBR Safe	
Loading buffer	
Ultra II End-prep reaction buffer	Nanopore library preparation
Ultra II End-prep enzyme mix	
AMPure XP beads	
Fresh 70% ethanol	
Adapter Mix	
Blunt/TA Ligation Master Mix	
Elution Buffer	
Loading primer	Nanopore loading
Fuel mix	
