# Supplementary material for: Real-time DNA barcoding in a rainforest using nanopore sequencing: opportunities for rapid biodiversity assessments and local capacity building
Source: Gigascience. 2018 Apr 2;7(4):giy033. doi: 10.1093/gigascience/giy033 (PMC5905381; doi:10.1093/gigascience/giy033)
Supplement: Supplemental material [file giy033_supp.zip › Supplementary Table 2.pdf]

Supplementary Table 2. Primers used in this study. Bold nucleotides indicate the universal tailed ONT sequences for the barcoding kit.

Locus	Primer name	Sequence (5’ - 3’)
16S	**16S_F_ONT**	**TTTCTGTTGGTGCTGATATTGC**CGCCTGTTTAYCAAAAACAT
	**16S_R_ONT**	**ACTTGCCTGTCGCTCTATCTTC**CCGGTCTGAACTCAGATCACGT
ND4	**ND4_F_ONT**	**TTTCTGTTGGTGCTGATATTGC**CACCTATGACTACCAAAAGCTCATGTAGAAGC
	**ND4_R_ONT**	**ACTTGCCTGTCGCTCTATCTTC**CATTACTTTTACTTGGATTTGCACCA
CytB	**CytB_F_ONT**	**TTTCTGTTGGTGCTGATATTGC**GACCTGTGATMTGAAAACCAYCGTTGT
	**CytB_R_ONT**	**ACTTGCCTGTCGCTCTATCTTC**CTTTGGTTTACAAGAACAATGCTTTA
