# Supplementary material for: Real-time DNA barcoding in a rainforest using nanopore sequencing: opportunities for rapid biodiversity assessments and local capacity building
Source: Gigascience. 2018 Apr 2;7(4):giy033. doi: 10.1093/gigascience/giy033 (PMC5905381; doi:10.1093/gigascience/giy033)
Supplement: Supplemental material [file giy033_supp.zip › Supplementary Table 4.pdf]

Supplementary Table 3. Subsampling report: impact of coverage on the consensus accuracy by randomly subsampling.

Subsampling scheme for viper

	Reads	Assembled reads	Mapped reads	Mismatches	Accuracy
**Set 1**	30	25	30	1 deletion poly T (5)	99.8%
	30	29	30	1 deletion poly T (6)	99.8%
	30	26	30	3 deletions poly T (5, 6, 5)	99.4%
**Set 2**	100	57	100	1 deletion poly T (5)	99.8%
	100	53	100	2 deletions poly T (5, 6)	99.6%
	100	62	100	-	**100%**
**Set 3**	300	60	300	1 deletion poly T (5)	99.8%
	300	62	300	1 deletion poly T (5)	99.8%
	300	53	300	1 deletion poly T (5)	99.8%
**Set 4**	1000	29	1000	1 deletion poly T (5)	99.8%
	1000	56	1000	1 deletion poly T (5)	99.8%
	1000	9	1000	-	**100%**

Subsampling scheme for gecko 1

	Reads	Assembled reads	Mapped reads	Mismatches	Accuracy
**Set 1**	30	30	30	3 deletions poly A/A/C (5/4/5)	99.4%
	30	17	30	2 deletions poly A/C (5/5)	99.6%
	30	15	30	1 insertion poly A (5), 1 deletions poly C (5)	99.8%
**Set 2**	100	62	100	one T instead of C (2Cs)	99.8%
	100	26	100	-	**100%**
	100	51	100	-	**100%**
**Set 3**	300	52	299	-	**100%**
	300	56	300	-	**100%**
	300	7	299	-	**100%**
**Set 4**	1000	1	999	1 C instead of T (CT)	99.8%
	1000	57	999	-	**100%**
	1000	57	998	-	**100%**
