# Supplementary figures and images for: Real-time DNA barcoding in a rainforest using nanopore sequencing: opportunities for rapid biodiversity assessments and local capacity building
Source: Gigascience. 2018 Apr 2;7(4):giy033. doi: 10.1093/gigascience/giy033 (PMC5905381; doi:10.1093/gigascience/giy033)

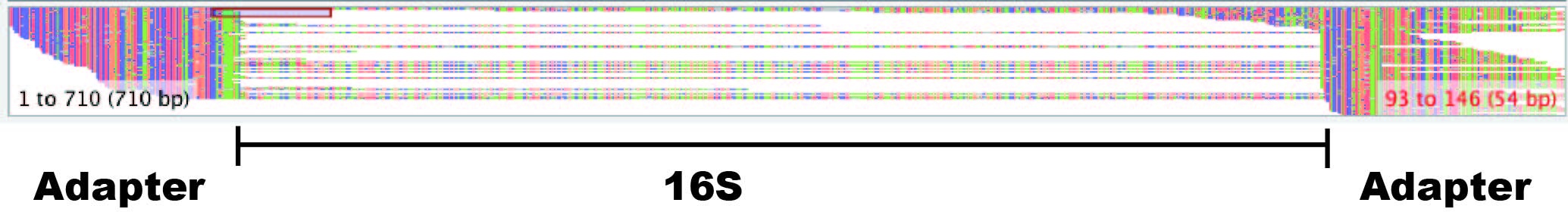

Supplement: Supplemental material [file giy033_supp.zip › Supplementary_Figure_1.jpg]

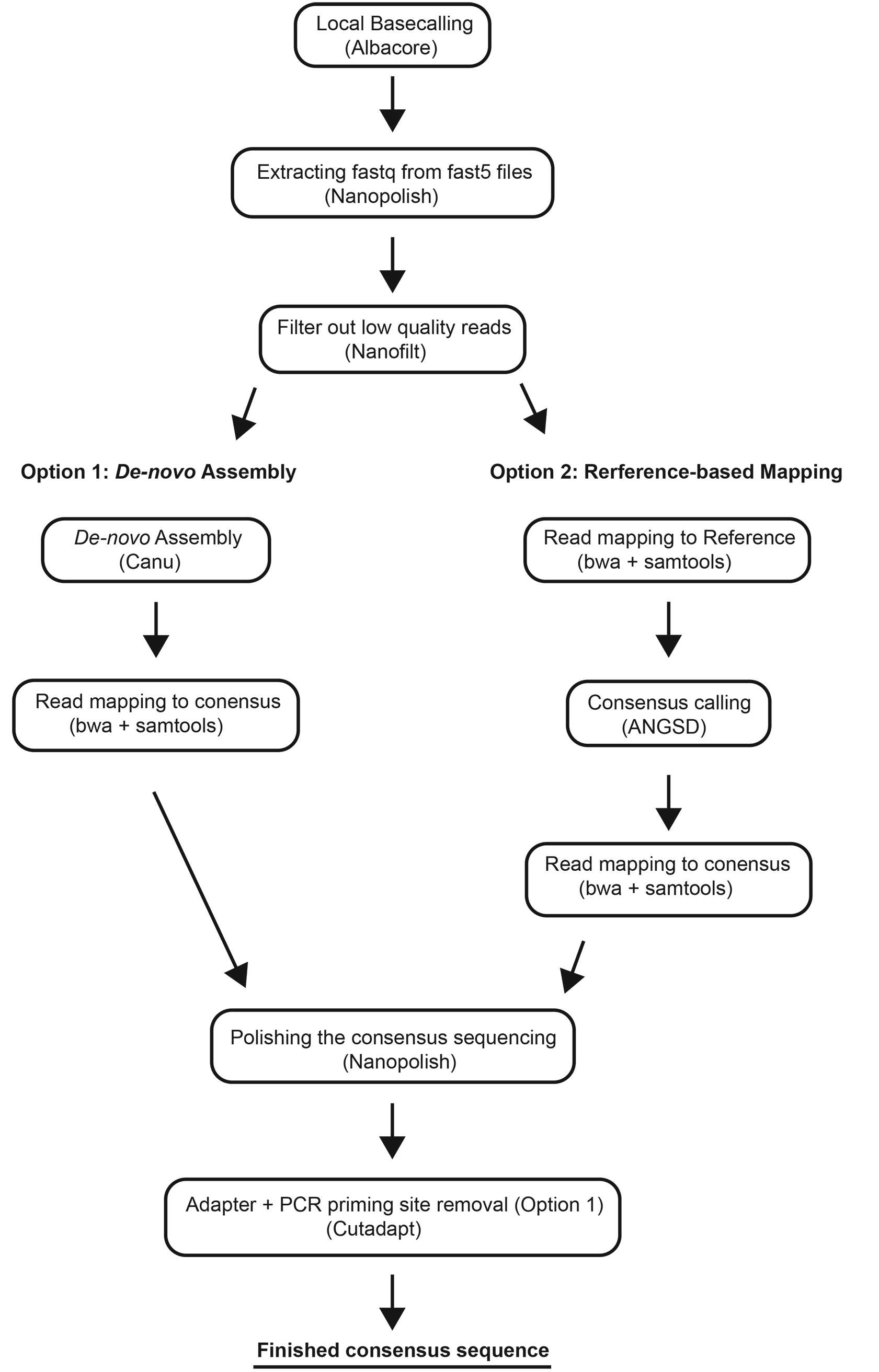

Supplement: Supplemental material [file giy033_supp.zip › Supplementary_Figure_2.tif]

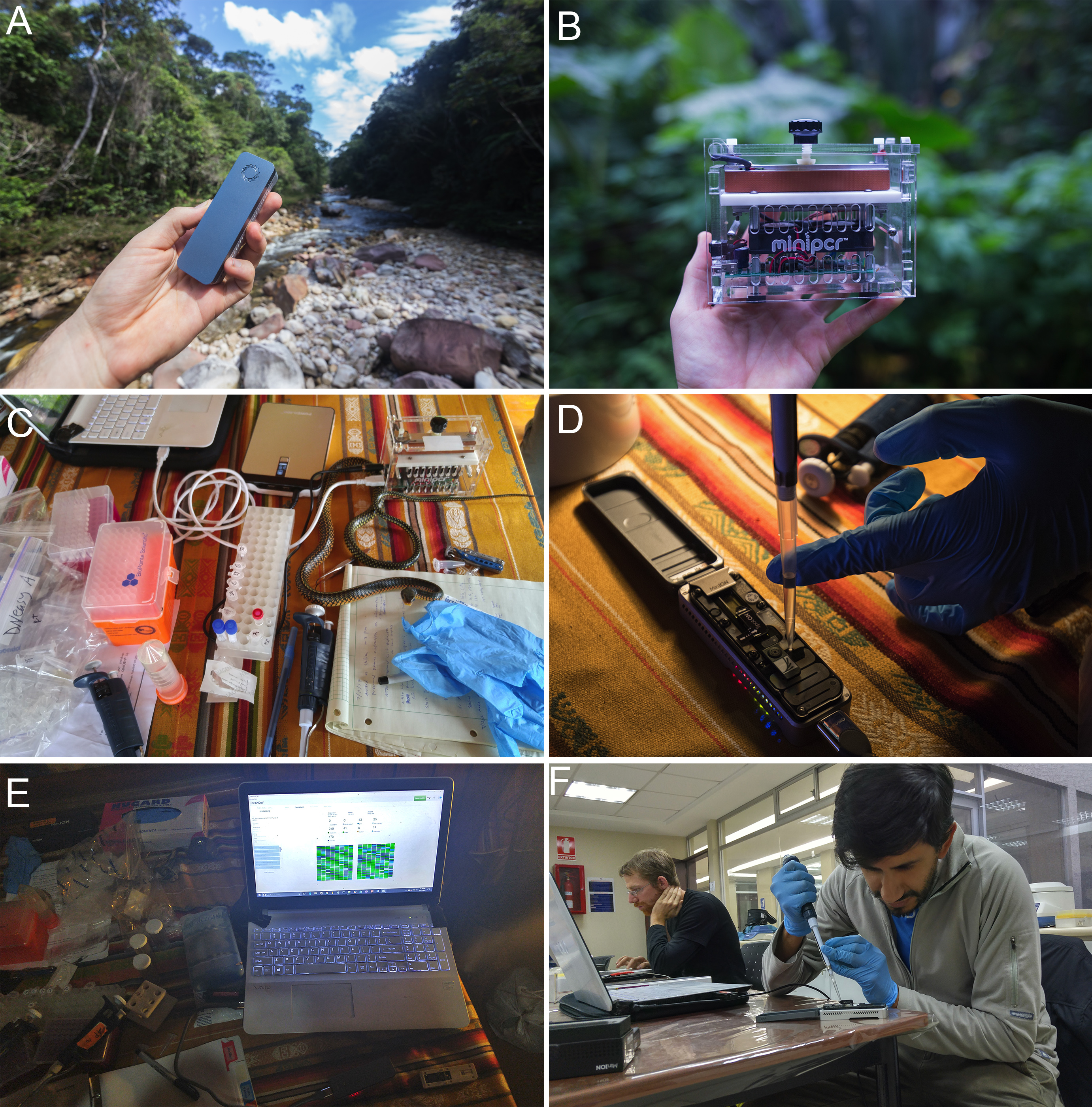

Supplement: Supplemental material [file giy033_supp.zip › Supplementary_Figure_3.jpg]

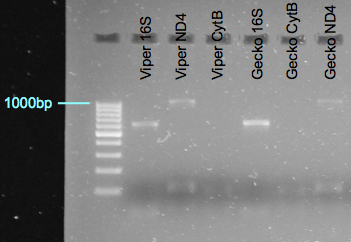

Supplement: Supplemental material [file giy033_supp.zip › Supplementary_Figure_4.jpg]
