# Supplementary material for: Real-time DNA barcoding in a rainforest using nanopore sequencing: opportunities for rapid biodiversity assessments and local capacity building
Source: Gigascience. 2018 Apr 2;7(4):giy033. doi: 10.1093/gigascience/giy033 (PMC5905381; doi:10.1093/gigascience/giy033)
Supplement: Response_to_Reviewer_Comments_Original_Submission.pdf [file giy033_response_to_reviewer_comments_original_submission.pdf]

Author's Response To Reviewer Comments

00000000-0000-(	VKSjlxg0Rb9gw H
E2A9317A	G96d6/NC8yKOv

Close

Reviewer reports:

Reviewer #1: The authors show us the possibility of real time DNA barcoding by utilizing a few portable laboratory fittings, especially the MinION sequencing platform, which is one of several sequencers that is small enough to be portable for field work. As the authors may aware that several studies have already published in respect to such interests, e. g. Michele et al, Plos One, 2017, on site DNA barcoding by nanopore sequencing.
In addition, the authors may want to conduct some extra analyses and show more details of their results to reach several of their main conclusions. For example:

1. It asked a lot of work to figure out how many samples on earth have been proceeded in current study, which of them have been processed in field? Have all of them conducted PCR aiming at 3 targeted amplicons? All the information can be easily and succinctly demonstrated in one table.

Thank you for the suggestion, we have added a supplementary table that includes all samples from the two runs. This should make it much easier for readers to see what sequencing was performed.

2. By subsampling analysis, the authors tried to say a minimum 30 reads are enough to produce reliable barcode sequences, however, the cytb has got > 30 reads while failed to achieve the final consensus sequence. The other markers can also conduct subsampling of 30 reads at least. In addition, the high throughput sequencing platforms are more sensitive and can generate reads for PCR amplicons albeit no clear band via gel electrophoresis. So, the authors should offer more evidences to say that "none of the reads actually belongs to CytB".

We blasted all reads and added the results to the manuscript. The analysis showed that we only retrieved 0 to ~3 reads for cytB for all samples. We think that the PCR settings, while working well for 16S, did not perform as well for cytB, and have discussed this in the text.

3. The sequencing and Bioinformatics section in supplementary information contains quite a lot valuable information that can help readers better understand current study, the authors may want to move some of the key info to main text. The authors may want to provide details in the form of a table regarding to, for example, how many reads obtained for each marker of each species, the number left after quality filtering, their corresponding length distribution, et al.

We moved the supplementary sections into the main manuscript, and shortened some sections to reduce the length and make it easier to read. We also added % of endogenous DNA barcode reads and % of adapters to the Supplementary table.

4. The authors tried to attribute the higher error rate of ND4 to contamination. Nanopore reads have extremely high error rate, especially for 1D reads, so it won't be a surprise for me if the reads got a top hit to another taxonomic group on NCBI. So, it is better to blast the ND4 reads of another species, e.g. Gecko 2 in your study, to NCBI to see what you can get before reaching the conclusion of contamination. BTW, I suppose you constructed 1D library rather than 2D, please
provide such info in your text.

We blasted ND4 for all species against NCBI, we moved that information from the Supplementary to the main manuscript. As can be seen in the new Supplementary table, the Gecko 1 ND4 reads have a much lower % of endogenous ND4 reads when compared to the viper sample (10% compared to 70%).

5. ANGSD -dofasta algorithm would not introduce indel to references, which can be one of the reasons why some of your reference-based consensus sequences contained more errors. The authors may want to compare the references, angsd-based consensus and their corresponding sanger sequences to see whether the discrepancies between each other will engender such bias.

In many cases we found the differences to be insertions of >2 bp compared to the Sanger sequence. In general, mapping approaches show a reference bias, especially for longer indels, but we did not find any of those in our sequenced samples.

6. Fig. 2: the 2 options share the same "read mapping to consensus" that can be merged. Plus, it would be better to provide more details of the denovo method, for example, did you choose the assemblies containing the highest number of reads as candidate consensus? If so, one of the subsampling set 4 (1000 reads, table S3) of gecko 1 got its assembly with only 1 read, what's your criteria for the following selection? And the authors also mentioned that "While the CytB de novo assembly did not succeed (no two reads assembled together)". Did the authors adopt exact same criteria all through?

We changed Supplementary Figure 2. We also added the requested info to the methods.

7. The second sequencing run at UTI generated quite a few adapter dimmers, how about that in the first run? Have you checked that? The information provided in Fig. S1 is quite bewildering, what does the length represent for, 710 bp and 54 bp? Also, what does the red rectangle represent for? Plus, what's the length of adapters, can they be filtered out in the very first step.

We removed Supplementary Figure 1, and added the info to the new Supplementary table. We used filtering using size to establish the % of adapters. So, yes it could be used for filtering, but since the adapter sequences are removed in the processing anyway, it should not matter whether they are removed or not.

8. When the authors talked about cost, not all your amplicons have been successfully sequenced, so $45 per barcode is far from correct estimation. and higher demultiplexing (>300) means less reads generated for each index, which will inevitably lead to higher error rate. The authors may want to tone down this part. In addition, several studies have tried to get individual barcoding at low cost using Miseq and Hiseq platform, you may want to add them in your citation, for example,
Shokralla S, Porter TM, Gibson JF et al. Massively parallel multiplex DNA sequencing for specimen identification using an Illumina MiSeq platform. Sci Rep 2015;5.

Meier R, Wong W, Srivathsan A et al. $1 DNA barcodes for reconstructing complex phenomes and finding rare species in specimen-rich samples. Cladistics 2016;32(1):100-10.

Liu, S., Yang, C., Zhou, C., & Zhou. Filling reference gaps via assembling DNA barcodes using high-throughput sequencing - moving toward barcoding the world. GigaScience, 2017; 1-8.

We have incorporated this additional information to the manuscript. Since we only sequenced a small subset of the total bases of what the MinION can produce there is no issue with low coverage for multiplexing > 300bp. New flow cells reliably produce > 10Gb of output, so comparable output to the MiSeq. We added information on Illumina barcoding to the text.

Lines 195 - 196: it would be better to provide the length of targeted amplions here.
Line 227: What's CS control?
Line 292: Fig. S4 ?
Line 473: please provide ratio at the same time

Additional suggestions/questions addressed in text.

Reviewer #2: Pomerantz et al describe the use of portable nanopore sequencing to assess biodiversity and identify animal species. The paper describes a consequential advance in that portable biodiversity assessment offers a powerful tool for conservation, research and education. I consider the approach of the authors rigourous and the presentation of data and advance made clear.

My major concern is that the paper spends approximately 250 out of the 600 lines in the main body of the manuscript discussing the broader implications of the work described, rather than the work itself. Evidently, this is an important element of a discussion section, but the narrative developed from ca. line 350 towers over the actual work performed. Many areas of this narrative are speculative: an important role of the discussion is to evaluate the work performed itself, placing it into context, rather than flagging all the possible areas of potential use, or cost implications (which will likely change rapidly) I would strongly recommend that the results and discussion are seperated, and that the discussion is condensed significantly, to strengthen its focus on the results, their limitations and interpretations - before a briefer subsection of the discussion deals with the broader interest areas. This is likely to necessitate a substantial rewrite of the results/discussion section.

We have moved the results from the Supplementary to the main text, thank you for the suggestion. We have also condensed parts of the text, including in the introduction and discussion.

Figure 1 is of contextual interest only and is not required to understand the advance presented. It should be removed or merged with Figure 2, which is generally illustrative of the research process.

Genbank data is listed as pending - thanks for making at available for review.

Minor comments.
L116: Minor point: Not necessarily a standard laptop computer. The specification of laptop according to Oxford Nanopore Technologies is very high, constraining accessiblity.
L121: Snowdonia National Park, Wales
L123-126: this can be updated to include bacterial ID on the ISS.
L278: Should this read Results & Discussion?

Additional suggestions have been incorporated in the text.

Close
