# Supplementary material for: Real-time DNA barcoding in a rainforest using nanopore sequencing: opportunities for rapid biodiversity assessments and local capacity building
Source: Gigascience. 2018 Apr 2;7(4):giy033. doi: 10.1093/gigascience/giy033 (PMC5905381; doi:10.1093/gigascience/giy033)
Supplement: Response_to_Reviewer_Comments_Revision_1.pdf [file giy033_response_to_reviewer_comments_revision_1.pdf]

Author's Response To Reviewer Comments

00000000-0000-(	609GLRp43HPw u
E2A9317A	zMm2OIg5M/4/Ae

Close

As per reviewer #2's suggestion, we have removed Figure 1 and adjusted the text accordingly.

Close
