# Supplementary material for: Real-time DNA barcoding in a rainforest using nanopore sequencing: opportunities for rapid biodiversity assessments and local capacity building
Source: Gigascience. 2018 Apr 2;7(4):giy033. doi: 10.1093/gigascience/giy033 (PMC5905381; doi:10.1093/gigascience/giy033)
Supplement: Reviewer_1_Report_(Original_Submission) -- Shanlin Liu — 1/4/2018 Reviewed [file giy033_reviewer_1_report_(original_submission).pdf]

Reviewer Report

Title: Real-time DNA barcoding in a rainforest using nanopore sequencing: opportunities for rapid biodiversity assessments and local capacity building

Version: Original Submission **Date:** 1/4/2018

Reviewer name: Shanlin Liu

Reviewer Comments to Author:

The authors show us the possibility of real time DNA barcoding by utilizing a few portable laboratory fittings, especially the MinION sequencing platform, which is one of several sequencers that is small enough to be portable for field work. As the authors may aware that several studies have already published in respect to such interests, e. g. Michele et al, Plos One, 2017, on site DNA barcoding by nanopore sequencing.
In addition, the authors may want to conduct some extra analyses and show more details of their results to reach several of their main conclusions. For example:

1. It asked a lot of work to figure out how many samples on earth have been proceeded in current study, which of them have been processed in field? Have all of them conducted PCR aiming at 3 targeted amplicons? All the information can be easily and succinctly demonstrated in one table.
2. By subsampling analysis, the authors tried to say a minimum 30 reads are enough to produce reliable barcode sequences, however, the cytb has got > 30 reads while failed to achieve the final consensus sequence. The other markers can also conduct subsampling of 30 reads at least. In addition, the high throughput sequencing platforms are more sensitive and can generate reads for PCR amplicons albeit no clear band via gel electrophoresis. So, the authors should offer more evidences to say that "none of the reads actually belongs to CytB".
3. The sequencing and Bioinformatics section in supplementary information contains quite a lot valuable information that can help readers better understand current study, the authors may want to move some of the key info to main text. The authors may want to provide details in the form of a table regarding to, for example, how many reads obtained for each marker of each species, the number left after quality filtering, their corresponding length distribution, et al.
4. The authors tried to attribute the higher error rate of ND4 to contamination. Nanopore reads have extremely high error rate, especially for 1D reads, so it won't be a surprise for me if the reads got a top hit to another taxonomic group on NCBI. So, it is better to blast the ND4 reads of another species, e.g. Gecko 2 in your study, to NCBI to see what you can get before reaching the conclusion of contamination. BTW, I suppose you constructed 1D library rather than 2D, please provide such info in your text.
5. ANGSD -dofasta algorithm would not introduce indel to references, which can be one of the reasons why some of your reference-based consensus sequences contained more errors. The authors may want to compare the references, angsd-based consensus and their corresponding sanger sequences to see whether the discrepancies between each other will engender such bias.
6. Fig. 2: the 2 options share the same "read mapping to consensus" that can be merged. Plus, it would be better to provide more details of the denovo method, for example, did you choose the assemblies containing the highest number of reads as candidate consensus? If so, one of the subsampling set 4 (1000 reads, table S3) of gecko 1 got its assembly with only 1 read, what's your criteria for the following selection? And the authors also mentioned that "While the CytB de novo assembly did not succeed (no two reads assembled together)". Did the authors adopt exact same criteria all through?
7. The second sequencing run at UTI generated quite a few adapter dimmers, how about that in the first run? Have you checked that? The information provided in Fig. S1 is quite bewildering, what does the length represent for, 710 bp and 54 bp? Also, what does the red rectangle represent for? Plus, what's the length of adapters, can they be filtered out in the very first step.

8. When the authors talked about cost, not all your amplicons have been successfully sequenced, so $45 per barcode is far from correct estimation. and higher demultiplexing (>300) means less reads generated for each index, which will inevitably lead to higher error rate. The authors may want to tone down this part. In addition, several studies have tried to get individual barcoding at low cost using Miseq and Hiseq platform, you may want to add them in your citation, for example,
Shokralla S, Porter TM, Gibson JF et al. Massively parallel multiplex DNA sequencing for specimen identification using an Illumina MiSeq platform. Sci Rep 2015;5.
Meier R, Wong W, Srivathsan A et al. $1 DNA barcodes for reconstructing complex phenomes and finding rare species in specimen-rich samples. Cladistics 2016;32(1):100-10.
Liu, S., Yang, C., Zhou, C., & Zhou. Filling reference gaps via assembling DNA barcodes using high-throughput sequencing - moving toward barcoding the world. GigaScience, 2017; 1-8.

Lines 195 - 196: it would be better to provide the length of targeted amplions here.
Line 227: What's CS control?
Line 292: Fig. S4 ?
Line 473: please provide ratio at the same time

Methods

Are the methods appropriate to the aims of the study, are they well described, and are necessary controls included? Yes

Conclusions

Are the conclusions adequately supported by the data shown? No

Reporting Standards

Does the manuscript adhere to the journal's guidelines on minimum standards of reporting? Yes

Choose an item.

Statistics

Are you able to assess all statistics in the manuscript, including the appropriateness of statistical tests used? There are no statistics in the manuscript.

Quality of Written English

Please indicate the quality of language in the manuscript: Acceptable

Declaration of Competing Interests

Please complete a declaration of competing interests, considering the following questions:

- Have you in the past five years received reimbursements, fees, funding, or salary from an organisation that may in any way gain or lose financially from the publication of this manuscript, either now or in the future?
- Do you hold any stocks or shares in an organisation that may in any way gain or lose financially from the publication of this manuscript, either now or in the future?
- Do you hold or are you currently applying for any patents relating to the content of the manuscript?
- Have you received reimbursements, fees, funding, or salary from an organization that holds or has applied for patents relating to the content of the manuscript?
- Do you have any other financial competing interests?
- Do you have any non-financial competing interests in relation to this paper?

If you can answer no to all of the above, write 'I declare that I have no competing interests' below. If your reply is yes to any, please give details below.

I declare that I have no competing interest

I agree to the open peer review policy of the journal. I understand that my name will be included on my report to the authors and, if the manuscript is accepted for publication, my named report including any attachments I upload will be posted on the website along with the authors' responses. I agree for my report to be made available under an Open Access Creative Commons CC-BY license (http://creativecommons.org/licenses/by/4.0/). I understand that any comments which I do not wish to be included in my named report can be included as confidential comments to the editors, which will not be published.

I agree to the open peer review policy of the journal

To further support our reviewers, we have joined with Publons, where you can gain additional credit to further highlight your hard work (see: https://publons.com/journal/530/gigascience). On publication of this paper, your review will be automatically added to Publons, you can then choose whether or not to claim your Publons credit. I understand this statement.

Yes Choose an item.
