# Supplementary material for: Real-time DNA barcoding in a rainforest using nanopore sequencing: opportunities for rapid biodiversity assessments and local capacity building
Source: Gigascience. 2018 Apr 2;7(4):giy033. doi: 10.1093/gigascience/giy033 (PMC5905381; doi:10.1093/gigascience/giy033)
Supplement: Reviewer_2_Report_(Original_Submission) -- Arwyn Edwards — 1/11/2018 Reviewed [file giy033_reviewer_2_report_(original_submission).pdf]

**Reviewer Report**

**Title: Real-time DNA barcoding in a rainforest using nanopore sequencing: opportunities for rapid biodiversity assessments and local capacity building**

**Version: Original Submission** **Date:** 1/11/2018

**Reviewer name: Arwyn Edwards**

**Reviewer Comments to Author:**

Pomerantz et al describe the use of portable nanopore sequencing to assess biodiversity and identify animal species. The paper describes a consequential advance in that portable biodiversity assessment offers a powerful tool for conservation, research and education. I consider the approach of the authors rigourous and the presentation of data and advance made clear.

My major concern is that the paper spends approximately 250 out of the 600 lines in the main body of the manuscript discussing the broader implications of the work described, rather than the work itself. Evidently, this is an important element of a discussion section, but the narrative developed from ca. line 350 towers over the actual work performed. Many areas of this narrative are speculative: an important role of the discussion is to evaluate the work performed itself, placing it into context, rather than flagging all the possible areas of potential use, or cost implications (which will likely change rapidly) I would strongly recommend that the results and discussion are seperated, and that the discussion is condensed significantly, to strengthen its focus on the results, their limitations and interpretations - before a briefer subsection of the discussion deals with the broader interest areas. This is likely to necessitate a substantial rewrite of the results/discussion section.

Figure 1 is of contextual interest only and is not required to understand the advance presented. It should be removed or merged with Figure 2, which is generally illustrative of the research process.

Genbank data is listed as pending - thanks for making at available for review.

Minor comments.
L116: Minor point: Not necessarily a standard laptop computer. The specification of laptop according to Oxford Nanopore Technologies is very high, constraining accessiblity.
L121: Snowdonia National Park, Wales
L123-126: this can be updated to include bacterial ID on the ISS.
L278: Should this read Results & Discussion?

**Methods**

Are the methods appropriate to the aims of the study, are they well described, and are necessary controls included? Yes

**Conclusions**

Are the conclusions adequately supported by the data shown? Yes

**Reporting Standards**

Does the manuscript adhere to the journal's guidelines on [minimum standards of reporting?](#) Yes

Choose an item.

**Statistics**

Are you able to assess all statistics in the manuscript, including the appropriateness of statistical tests used? Yes, and I have assessed the statistics in my report.

**Quality of Written English**

Please indicate the quality of language in the manuscript: Acceptable

**Declaration of Competing Interests**

Please complete a declaration of competing interests, considering the following questions:

- Have you in the past five years received reimbursements, fees, funding, or salary from an organisation that may in any way gain or lose financially from the publication of this manuscript, either now or in the future?
- Do you hold any stocks or shares in an organisation that may in any way gain or lose financially from the publication of this manuscript, either now or in the future?
- Do you hold or are you currently applying for any patents relating to the content of the manuscript?
- Have you received reimbursements, fees, funding, or salary from an organization that holds or has applied for patents relating to the content of the manuscript?
- Do you have any other financial competing interests?
- Do you have any non-financial competing interests in relation to this paper?

If you can answer no to all of the above, write 'I declare that I have no competing interests' below. If your reply is yes to any, please give details below.

Costs for attending and presenting at the Nanopore Community Meeting in New York in 2016 and 2017 have been covered by Oxford Nanopore Technologies, Ltd. Oxford Nanopore Technologies Ltd have supplied free reagents for outreach work in September 2017. No direct funding, fees or salary has been provided, and there are no other competing interests to declare.

I agree to the open peer review policy of the journal. I understand that my name will be included on my report to the authors and, if the manuscript is accepted for publication, my named report including any attachments I upload will be posted on the website along with the authors' responses. I agree for my report to be made available under an Open Access Creative Commons CC-BY license (http://creativecommons.org/licenses/by/4.0/). I understand that any comments which I do not wish to

be included in my named report can be included as confidential comments to the editors, which will not be published.

I agree to the open peer review policy of the journal

To further support our reviewers, we have joined with Publons, where you can gain additional credit to further highlight your hard work (see: https://publons.com/journal/530/gigascience). On publication of this paper, your review will be automatically added to Publons, you can then choose whether or not to claim your Publons credit. I understand this statement.

Yes Choose an item.
